# Exploring Flow Procedures for Diazonium Formation

**DOI:** 10.3390/molecules21070918

**Published:** 2016-07-14

**Authors:** Te Hu, Ian R. Baxendale, Marcus Baumann

**Affiliations:** Department of Chemistry, University of Durham, South Road, Durham DH1 3LE, UK; te.hu@durham.ac.uk (T.H.); marcus.baumann@durham.ac.uk (M.B.)

**Keywords:** diazonium salts, flow chemistry, meso reactor, processing, supported reagent

## Abstract

The synthesis of diazonium salts is historically an important transformation extensively utilized in dye manufacture. However the highly reactive nature of the diazonium functionality has additionally led to the development of many new reactions including several carbon-carbon bond forming processes. It is therefore highly desirable to determine optimum conditions for the formation of diazonium compounds utilizing the latest processing tools such as flow chemistry to take advantage of the increased safety and continuous manufacturing capabilities. Herein we report a series of flow-based procedures to prepare diazonium salts for subsequent in-situ consumption.

## 1. Introduction

The formation and continuous processing of highly reactive or potentially unstable intermediates has proven to be a strong driver for the adoption of flow based chemical synthesis [1,2,3]. Indeed, the ability to continuously prepare transient species using small volume reactor technology and directly couple their formation into a subsequent consuming reaction step has significantly enhanced the safety profile of many chemical sequences [4,5,6,7]. For this reason we are currently experiencing a resurgence of interest in many classical transformations that have historically been relegated to almost obscurity because of inherent batch based safety concerns and an inability to scale the transformation [8,9,10,11,12,13,14,15,16]. The improved mixing efficiencies and greater temperature control imparted through the application of flow based reactor technologies is thus enabling their reinvestigation. From our own repertoire of studies the diazonium functionality has shown particular versatility as a reactive intermediate [17,18,19,20] that benefits from being prepared and directly reacted in a continuous flowing process [21,22,23,24,25,26,27,28,29,30,31,32,33,34,35,36,37]. Within this manuscript we describe a number of methods that can be used to conveniently prepare these species at differing scale and with contrasting processing advantages.

## 2. Results and Discussion

We initially started our investigations by evaluating the various methods of forming aryl diazonium salts under flow conditions. These can be broadly classified into three general methods based upon the phases used, namely: aqueous (Section 2.1); organic (Section 2.2) and solid phase (Section 2.3).

### 2.1. Formation of Aryl Diazonium Species under Aqueous Conditions

**Figure 1:**
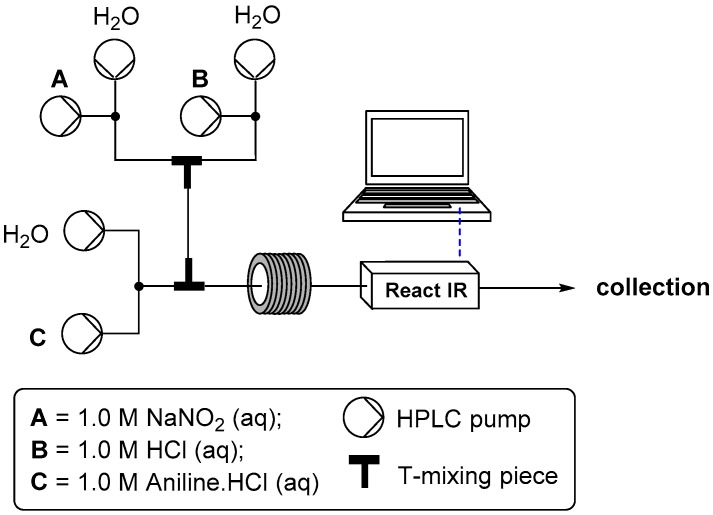
Configuration of flow reactors for diazonium formation using aqueous conditions.

For the development of the aqueous conditions for preparing diazonium salts in flow we evaluated the classical combination of sodium nitrite and hydrochloric acid. To aid in the rapid optimization of the transformation we incorporated flow ReactIR analysis into the process [38,39]. The reactor setup consisted of six paired HPLC pumps used to deliver three variable concentrations of the different reaction inputs (Figure 1). Stream one contained the sodium nitrite solution, stream two an aqueous solution of hydrochloric acid and stream three the aniline component as its mono-hydrochloric acid salt [40], also dissolved in water. The final configuration of T-mixing pieces as shown in Figure 1 was determined through experimental testing. It was found that mixing the hydrochloric acid and nitrite stream prior to introduction of the aniline gave consistently superior results [41]. It was further observed that altering the delay time between this initial mixing event and subsequent introduction of the aniline stream had no detectable effect on the tested reactions (delay range 1.2–40 s). In this regard the aqueous acid catalyzed decomposition of inorganic nitrite to nitrous acid and further to nitric oxide is known to be a very rapidly established equilibrium process [42]. The presence of very low levels of a NO triple bonded species as determined by ReactIR at 2190–2215 cm^−1^ was noted, but it was not deemed useful to quantify its formation (generation of calibration curves) under these equilibrium conditions. Instead monitoring of the subsequent diazonium formation step was examined instead.

To test the range of viable reactant concentrations, 1.0 M stock solutions of the three reagents were prepared; water was used as the diluent for the three makeup pumps. Serial dilution profiles for each reagent stream were systematically produced whilst always ensuring the required theoretical minimum 1:1:1 reagent stoichiometry. It was quickly found that a viable concentration window of 0.2 + 0.25 M aniline, 1.8 equivalents of HCl and 1.3 equivalents of sodium nitrite worked well across a representative subset of anilines (Ar = 4-Me, 2-OMe, 2-F, 4-OMe, 4-Br, 3-NO_2_, 4-NO_2_ and 3-CF_3_). In general higher aniline concentration led to precipitation issues and significant by-product formation (triazine formation with low acid concentration). The use of 1.8 equivalents of hydrochloric acid gave excellent conversions to the diazonium salt in every case (>93% purity), however, using a higher ratio for the more electron rich anilines (Ar = 4-OMe {2.2 M} and 2-OMe {2.0 M}) was found to completely suppress the formation of small quantities of diazo coupled material and other unidentified side products [43,44,45,46,47,48,49]. Importantly, it should be noted that in each instance the first equivalent of the hydrochloric acid was always present as a part of the aniline stock solution (HCl salt), facilitating solubility of the aniline substrate in the aqueous media. Therefore only the further excess (>1 equivalent) is supplied as a separate stream and used in the generation of the reactive nitrosonium cation prior to its combination with the aniline flow stream (Figure 1). This is important because as identified earlier it was found that pre-generation of the intermediate NO+ containing solution prior to its unification with the aniline yielded improved results. This was additionally confirmed by altering the reaction setup to eliminate the separate acid stream and alternatively supply the same quantity of hydrochloric acid as a homogeneous mixture with the aniline starting material. The solutions thus generated although containing only the diazonium intermediate as the primary constituent (by ^1^H-NMR, ^13^C-NMR and HR-MS analysis) were definitively of lower purity [41]. In addition using these solutions as feeds for the subsequent steps also resulted in lower overall recovery of the desired addition products (see later description).

#### 2.1.1. Temperature Dependence

We next evaluated temperature dependence as a parameter of the reaction outcome. Normally temperatures close to 0 °C are employed in batch diazotization reactions to regulate the exothermic nature of the diazonium salt formation. Our objective was to determine if an operational window closer to ambient (25 °C) could be feasibly used to simplify the chemical processing in flow. It was initially found difficult to quantify the outcome of this assessment. Ultimately, it was found beneficial to compare the effect of temperature on the product output when calibrated against an internal standard. For this purpose we used 2-nitro-5-methoxytoluene (25 mol % wrt aniline) which provided simple calibration using both NMR and ReactIR analysis (placed in the aniline stock solution during processing).

To facilitate investigation of the diazonium stability at various temperatures a Polar Bear Plus flow synthesizer [50] was integrated into the system using pre-cooling incubation loops to regulate the temperature of the input fluids prior to mixing (set at the reactor temperature). It was rapidly determined that even at −10 °C the formation of the diazonium salt occurred rapidly and was complete for all evaluated substrates in less than 2.5 min residence time (flow rate 2.0 mL/min; 0.35 M aniline Ar = 4-NO_2_, 4-MeO and Ph, 1.8 equivalents of HCl and 1.3 equivalents of NaNO_2_).

**Figure 2:**
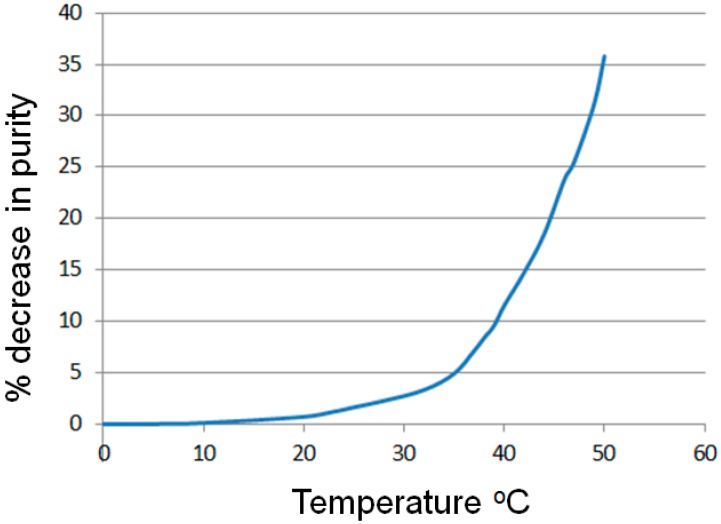
Plot of the percentage decrease in phenyl diazonium output against a change in temperature for phenyl diazonium.

An expanded temperature range of −10 to 50 °C was therefore investigated using the same fixed flow rate and reagent concentration to assess stability of the diazonium salt (Figure 2). Only a small decrease in purity of the diazonium adduct was detected between −10 and 10 °C (Ar = Ph < 0.1%, no aniline starting material was detected). Raising the temperature over the next 15 °C gave a small but incremental increase in the amount of diazonium impurities, measured as a reciprocal decrease in the relative quantity of diazonium detected relative to the internal standard (Ar = Ph ≤ 1.7%). Further heating then resulted in much higher levels of decomposition and identification of the corresponding phenolic products (as determined by LC-MS). Figure 2 shows the extracted plot of the flow stream as monitored by ReactIR analysis comparing the internal standard against the level of phenyl diazonium salt during a continuous temperature ramp (0.2 °C·min^−1^; data points generated by the summation of 20 independent temperature stabilized scans). As can be seen, for temperatures up to 20 °C only minor decomposition occurs, however, a significant onset in the rate of decomposition is seen at around 33 °C. Therefore a compromise cooling solution for this system would be a working temperature of 8–10 °C coupled with a shorter segmentation time before subsequent reaction of the newly formed diazonium species.

As expected due to the electronic factors affecting the subsequent formation of the intermediate carbocation from the 4-NO_2_ and 4-MeO substituted aryl diazonium these species proved much more stable to decomposition [43,44,45,46,47,48,49]. For these substrates the onset of any detectable decomposition was first observed at >25 °C and was not significant (>0.5%) until >40 °C was attained. Conducting an investigation of the literature confirmed that *ortho*- and *meta*-substitution patterns exert a more pronounced effect on the rates of diazonium decomposition leading to the corresponding phenolic derivative. However, conversely such substitution then often retards the subsequent phenol/diazo coupling process meaning the overall rates of decomposition/consumption of the parent diazonium approximates the simple phenyl unit albeit the by-product distribution is significantly different [43,44,45,46,47,48,49].

In summary, diazonium formation occurred very rapidly, requiring less than 1 min to ensure complete conversion of the starting aniline in the flow reactor when a reaction temperature of 10 °C is employed. The stability of the diazonium product was determined to be temperature dependent but showed acceptable processing stability at temperatures ≤10 °C over short hold times <5 min.

#### 2.1.2. Flow Rate Analysis

**Figure 3:**
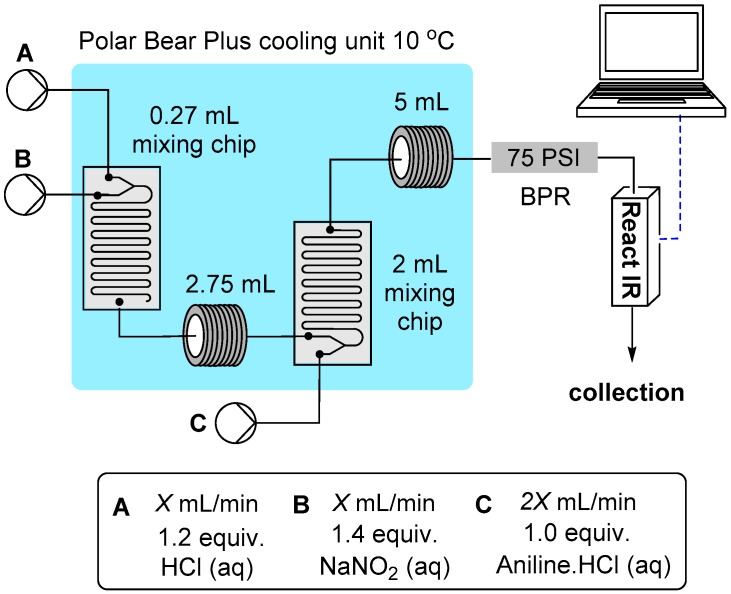
Flow reactor setup for diazotization at high flow rates.

Finally, an investigation into the viable flow rate range was undertaken. The rate determining step for the sequence is the formation of the *N*-nitrosoamine, however this is still extremely fast. Importantly we had already identified that product decomposition was only an issue as the reaction temperature approached ambient when using long diazonium hold times (see above discussion). Therefore a processing scenario involving fast throughputs seemed to offer an immediate advantage. Using a simplified three pump system we established the modified flow reactor setup as depicted in Figure 3. Initially at high flow rates we experienced poor mixing due to laminar flow resulting in incomplete conversion of the aniline starting material. This was quickly rectified by replacing the simple PEEK T-mixers in the system with dedicated mixing chips [51]. A 0.27 mL internal volume chip was used for the HCl and NaNO_2_ solution mixing and a larger 2 mL chip for further combining with the aniline solution at the higher combined flow rate. In addition a 2.75 mL cooling loop was placed directly after the first mixing chip and a 5 mL cooling coil after the second mixing chip. The entire mixing (chips) and reaction zone (tubular coils) was maintained at 10 °C using a single Polar Bear Plus flow synthesizer (a copper plate housing the two mixing chips was attached to the top of the Polar Bear’s central cooling column which also supported the 2.75 and 5 mL cooling coils). Using this setup even at high flow rates (X = 5 mL/min) at the limits of the HPLC pumping equipment (pump C operational maximum 10 mL/min) complete conversion to the diazonium salts was achieved across a spectrum of substrates; Ar = 4-Cl (**1**), 4-Me, 2-OMe, 4-OMe, 4-Br, 3-NO_2_ and 3-CF_3_. Furthermore, in each case a clean solution of the product as a yellow/orange liquid was produced without evidence of by-product formation. This was therefore adopted as the reactor setup for all future reactions under these aqueous conditions.

**Figure 4:**
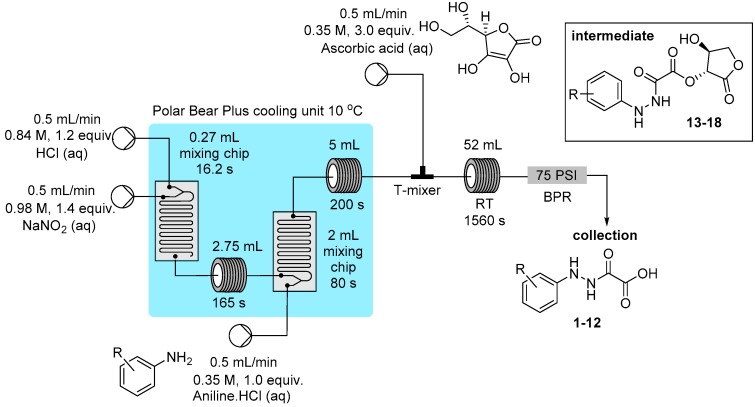
Flow preparation of masked hydrazine derivatives.

To exemplify the generation of the diazonium salts and the ability to trap in situ the reactive species in a linked flow process a small collection of oxamic acid derivatives were prepared through the reduction of the parent diazonium compound using solutions of ascorbic acid (Figure 4; total residence time of ~34 min) [52,53,54,55]. The final products **1**–**12** were easily isolated in very high purity using a batch extraction, involving a simple extraction following determination of the crude conversion (Table 1). It was also possible for certain substrates to isolate the intermediate ester (42%–62% yield) prior to hydrolysis (**13**–**18**: see Experimental Section), however in each case if the material was left to stand in the reaction mixture for >15 min the principle product in solution was the oxamic acid derivative. In practice the reactor output was left to stand for 1 h to ensure complete hydrolysis of the intermediate before work-up and isolation of the desired oxamic acid product.

### 2.2. Formation of Aryl Diazonium Species under Organic Non Aqueous Conditions

**Figure 5:**
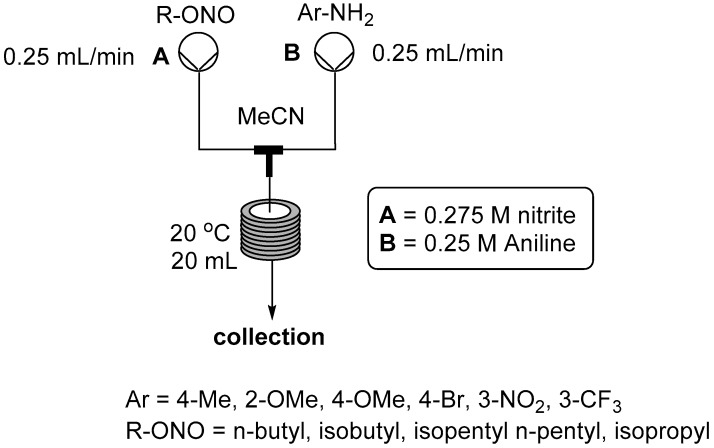
Flow reactor for diazonium formation under anhydrous conditions (material not isolated—anion not determined).

We have previously conducted several investigations into the preparation of diazonium salts as intermediates in organic solvents using alkyl nitrites as the corresponding NO+ source [17,18,19,20]. In our previous work we have employed almost exclusively *tert*-butyl nitrite as the diazotizing reagent due to its availability and higher safety profile for use in batch mode. However, several other materials exist which become viable alternatives representing a potential cost saving when employed at scale working safely within a flow regime. We therefore decided to test some of these other compounds; namely, *n*-butyl nitrite (CAS: 544-16-1), isobutyl nitrite (CAS: 542-56-3), isopentyl nitrite (CAS: 110-46-3), pentyl nitrite (CAS: 463-04-7) and isopropyl nitrite (CAS: 541-42-4); the reactor set-up used is shown in Figure 5. Conversion of the aniline starting material was determined by ^1^H-NMR analysis against an internal standard (2-nitro-5-methoxytoluene). Each of the tested reagents performed equally well using the general reactor setup as shown in Figure 4, although isopropyl nitrite proved more difficult to handle practically due to its low boiling point. In general it was found that *tert*-butyl nitrite could be directly substituted in this process without any change in observed yield or purity of the flow stream using 1.1 equivalents of the alkyl nitrite and a reaction temperature of 20 °C. It should be noted that a decreased concentration of the starting materials was needed due to the considerably lower solubility of several of the diazonium species in acetonitrile to avoid the issue of precipitation and blocking of the reactor.

**Figure 6:**
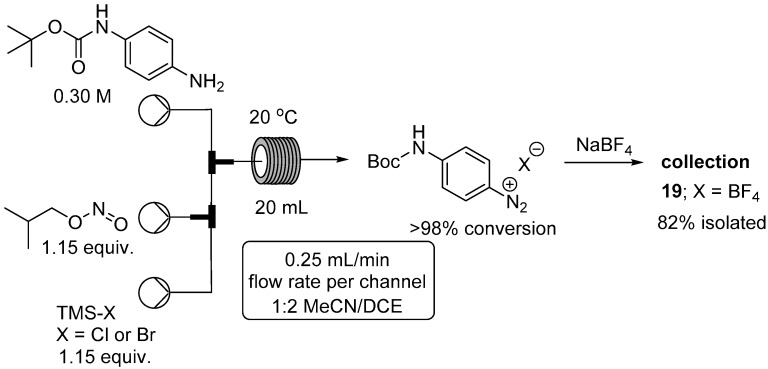
Flow reactor for diazotization of *tert*-butyl 4-aminophenylcarbamate.

We next considered the use of nitrosyl halides (BrNO and ClNO) which can also be used as diazotization reagents under anhydrous conditions [56]. These species are gases but can be readily generated in situ by the reaction of trimethylsilyl halides (Br/Cl) with alkyl nitrites, under essentially acid free conditions. This was highly desirable as we had experienced difficulties in achieving high isolated yields of the corresponding diazonium salt when using certain acid sensitive substrates. We therefore wished to evaluate these conditions for use with a particularly problematic substrate, namely *tert*-butyl 4-aminophenylcarbamate. Under our standard aqueous conditions we identified 16% doubly diazotized material and 53% mono-Boc protected diazonium product, the remaining being unidentified by-products [57]. We found the best reagent combination was 1.15 equivalents of isobutyl nitrite and trimethylsilyl halide in a 2:1 solvent mixture of dichloroethane and MeCN which gave good solubility of the diazonium salt (Figure 6). Satisfyingly under these conditions no doubly diazotized product was detected with only clean and complete conversion to the desired product being observed. To aid with safe isolation and enable full characterization the intermediate diazonium salt was converted to the tetrafluoroborate salt **19**. Due to the gaseous nature of the intermediate diazotizing agent we are currently working on their generation for use in other applications through the adoption of the flow tube-in-tube gas reactor system [58,59,60,61].

### 2.3. Formation of Aryl Diazonium Species Using Solid Phase Techniques

**Figure 7:**
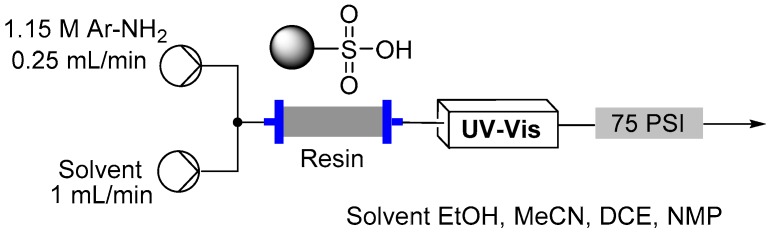
Flow reactor for diazonium formation under heterogeneous conditions.

In our previous flow work we have found it extremely beneficial to supplement packed cartridges of immobilized reagents into the flow streams to simplify the introduction of reagents or help purify reactions [62,63]. We were therefore interested in the potential of using such an approach to help generate diazonium salts in flow. The simplest approach we could envisage was to utilize a sulfonic acid functionalized ion exchange matrix to act as an acid source and to ultimately form a corresponding aryl-diazonium sulfate species retained by association to the support [64,65]. Interestingly, we found that a related strategy had already been adopted in batch using a sulfonic acid modified silica [66,67]. In addition we and Filimonov et al. had used a polymer bound nitrite source to assist in the formation of various arene-diazoniums [68,69]. Encouraged by these previous results we tested three support materials for the promotion of diazotization reactions in flow, namely, MP-TsOH [70], Si-SA (two forms namely SCX and SCX2) [71], and Nafion NR50 [72]. Glass Omnifit tubes were packed with the different solid supports and placed within the flow path of a Vapourtec R2+/R4 unit (Figure 7).

**Figure 8:**
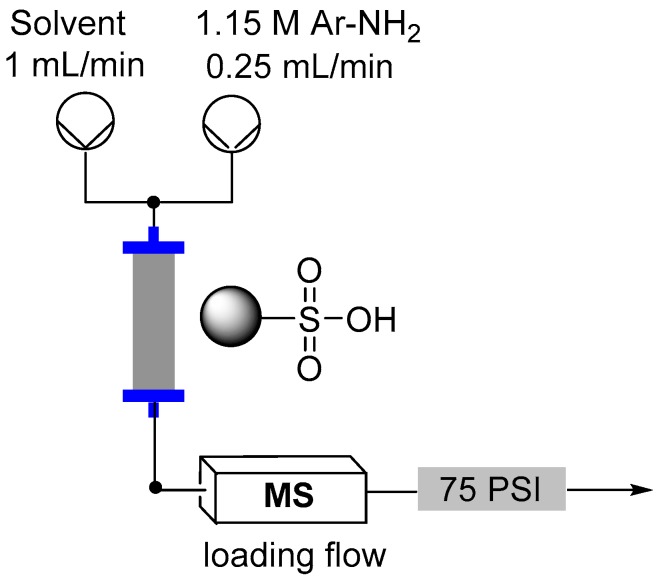
Flow reactor loading set-up.

Several loading approaches were contrasted for simplicity and efficiency. It was decided to pursue a non-aqueous set of reaction conditions because of the variable solubility of the different aniline starting materials at neutral pH in water. First, a solution of the aniline in an organic solvent (EtOH, MeCN, DCE or NMP; 1.15 M, flow rate 0.25 mL/min) was passed through the cartridge of immobilized acid. In general a discernible color change could be observed progressing through the cartridge as capture of the aniline occurred. To further assist with the determination of the extent of loading a UV-Vis detector (Gilson 155) was placed in-line to monitor for aniline breakthrough (scanning mode) [73]. Upon detection of aniline in the output line an automated valve trigger enabled exchange of the input flow to an alternative pure solvent stream. The column was washed using the pure solvent to remove any non-captured aniline, which could be collected for calibration of the loading and if required easily isolated for recycling. Again, this secondary washing process was monitored by UV-Vis to enable automated assessment and processing. Unfortunately, it was found that the results obtained were very inconsistent. Ultimately, it was shown that highly UV active impurities in the aniline starting materials were generating false positive detection signals resulting in premature termination of the loading sequence. To avoid this we instead repeated the loading but employed direct in-line MS analysis (Advion Expression CMS) enabling mass directed triggering [74,75]. This alternative approach allowed us to rapidly calibrate the active chemical loading of the sulfonic acid supports (Table 2). Beneficially the column washing stage could again be successfully conducted as previously stated monitoring for completeness using the in-line MS analysis. This therefore allowed us to progress a strategy of loading and washing in an automated fashion (Figure 8).

Analysis of Table 2. The relative differences between the theoretical and actual loading for the MP-TsOH and Nafion NR50 resins can be accounted for because of the degree of permeation of the aniline into the micro beads. The MP-TsOH is supplied as 375–575 micron particles, the silica supports as 40–63 µm spheres and the Nafion as large pellets (1.7–3 mm). With larger beads channeling effects can occur as the fluidic flow moves through the packed bed convecting the aniline through the cartridge and reducing the contact time for effective diffusion. In the case of the silica supports the smaller particle size and the fact the materials are only surface functionalized gives easy site access and rapid scavenging. This was shown by first doubling and then quadrupling the tested flow rate and examining the scavenging process (Table 2 data).

It should also be noted that changing the solvent from MeCN had a large impact on the capture results for the Nafion resin. Using NMP increased the sequestration rate whereas EtOH gave a correspondingly decrease. This can be rationalized by the expected swelling properties of the Nafion resin in these solvents changing the accessibility to the functional sites, NMP being a much better solvent for swelling the resin. The MP-TsOH being a highly cross-linked macroporous resin would not be affected to the same extent as the pores are more rigidly defined. This is consistent with the observed results.

In a further set of experiments it was also determined that the electronic properties of the aniline played a significant role in the scavenging efficiency of the species onto the solid supports. As expected weakly basic anilines such as those with nitro substituents required much longer contact times and often gave lower final loading capacity (Table 3). There was also a pronounced solvent effect with EtOH and NMP showing better uptake rates than MeCN and DCE or DCM. As the *p*Ka value of the corresponding parent solution phase *p*-toluene sulfonic acid is reported to be much higher in MeCN (8.5) than water (−2.8) or other hydrogen bonding solvents this is again consistent [76,77,78].

Although as previously shown reducing the flow through rate did increase the sequestering efficacy this was not general across all the anilines tested. It was found that even using a flow rate of 0.10 mL/min failed to increase the scavenging capacity >55% for weakly basic anilines such as the 2- or 4-NO_2_ functionalised species.

It was determined that in general this supported acid strategy could only be effectively used with the more intrinsically basic aniline substrates. It was therefore not possible to determine a generic set of loading conditions as significant individual optimization was required for each starting material. Preliminary attempts to establish a predictive model based upon a correlation between *p*Ka of the substrate and its loading were also not successful. Therefore although the loading sequence could be performed under automated control and it was possible to establish recycling of the aniline flow streams to increase loading capacities over time we felt this was not a practical approach to synthesis in this instance. However, having loaded a set of reagent columns with different anilines we proceeded to test the subsequent diazotization step as a proof of concept study.

A stock solution of isobutyl nitrite (1 M) was prepared in the corresponding aniline loading solvent and passed through each reactor cartridge in sequence. It should be noted that solvent swapping was successfully trialed and gave identical results to reacting in the original solvent, with the notable exception of the Nafion resin which was again put down to its swelling characteristics. Having experienced repeated problems with the Nafion resin we excluded this from any additional testing. Interestingly this capacity to exchange solvents offers an advantage for initially loading highly insoluble anilines (i.e., in NMP or mixed solvents) with the option of subsequently further processing them in a different solvent. In total two equivalents of isobutyl nitrite based upon the theoretical loading of the solid resin was directed through each column at a flow rate of 0.5 mL/min. A further 40 mL (~2.5 column volumes) of pure solvent at a flow rate of 1 mL/min was then used to wash the columns (wash mixture directed to waste).

**Figure 9:**
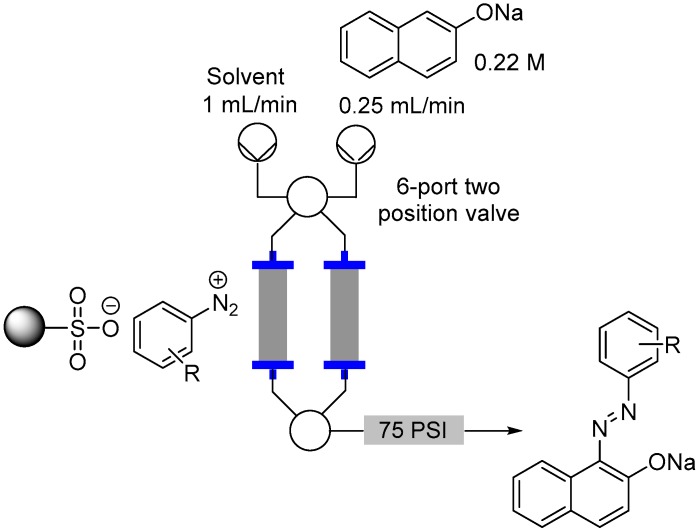
Flow reactor loading set-up.

To assess the quantity of diazotized species associated with the support we used a very facile diazo dye formation as mediated by reaction with a solution of 2-naphtholate (Figure 9). To this end a 0.22 M solution of sodium naphtholate prepared from equimolar quantities of sodium ethoxide and 2-naphthol was pumped through the reactor cartridge at a flow rate of 0.25 mL/min for 20 min. The reactor was then washed with EtOH and the output collected for a further 15 min to ensure complete elution of all the diazo species. From this combined output a 1 mL aliquot was sampled, evaporated and the residue redissolved in a standardized solution of *d*_6_-DMSO doped with a known concentration of 2-methoxytoluene. Subsequent calculation of the quantity of diazo dye was made by extrapolation from ^1^H-NMR assessment of the characteristic 1-naphthyl proton in the coupled product. The main bulk of output solution was evaporated, neutralized with 1 M HCl and extracted into EtOAc to allow isolation of the diazo product. Pleasingly the assessed conversions matched well with the previously determined loadings of the aniline starting materials (Table 3 shows the results for EtOH based on the use of MP-TsOH). In general a consistent but small decrease in the isolated yields of the diazo dye was observed verses the monitored loading of the resin (3%–12%). This demonstrated the high efficiency of the diazotization step on the solid phase. The notable outliers were substrates possessing strongly electron withdrawing nitro functionality which effects solubility and were notably difficult to fully elute from the column.

In summary, we have demonstrated that using a solid phase approach could be successfully applied to the formation and subsequent reaction of certain diazo species prepared from ionically immobilized anilines. This method offers certain advantages for direct in-line purification of these reactive intermediates and could add further value for reactions where solvent exchange or pH adjustment is required when reacting with the final diazonium species. However, a limiting aspect of this approach was found to be the initial loading of the anilines which was highly dependent upon their electronic nature. Although this could be assessed and the loading sequence could be achieved in an automated fashion this approach was deemed non-ideal for practical diazonium generation. As a result we would recommend the previously described aqueous and organic protocols as the main preparative methods moving forward.

## 3. Experimental Section

### 3.1. General Information

Unless otherwise stated, all solvents were purchased from Fisher Scientific (Bishop Meadow Rd, Loughborough, UK) and used without further purification. Substrates and reagents were purchased from Alfa Aesar (Shore Road, Lancashire, Heysham, UK) or Sigma Aldrich (New Road, Gillingham, Dorset, UK) and used as received.

^1^H-NMR spectra were recorded on either Avance-400 (Bruker, Elisabethhof 15, Leiderdorp, The Netherlands) or VNMRS-700 (Varian Medical Systems, Inc., 3100 Hansen Way, Palo Alto, CA, USA) instruments and peal positions are reported relative to residual solvent: CHCl_3_ (δ 7.26 ppm), DMSO-*d*_6_ (δ 2.50 ppm), MeOH-*d*_4_ (δ 3.31 ppm). ^13^C-NMR spectra were recorded on the same instruments and are reported relative to CHCl_3_ (δ 77.1 ppm), DMSO-*d*_6_ (δ 39.5 ppm) or MeOH-*d*_4_ (δ 49.0 ppm). Data for ^1^H-NMR are reported as follows: chemical shift (δ/ppm) (integration, multiplicity, coupling constant (Hz)). Multiplicities are reported as follows: s = singlet, d = doublet, t = triplet, q = quartet, p = pentet, m = multiplet, br. s = broad singlet, app = apparent. Data for ^13^C-NMR are reported in terms of chemical shift (δ/ppm) and multiplicity (C, CH, CH_2_ or CH_3_). Data for ^19^F-NMR were recorded on a Bruker Avance-400 instrument at a frequency of 376 MHz using CFCl_3_ as external standard. DEPT-135, COSY, HSQC, and HMBC experiments were used in the structural assignment. IR spectra were obtained by use of a RX1 spectrometer (Perkin Elmer, 940 Winter St., Waltham, MA, USA, neat, ATR sampling) with the intensities of the characteristic signals being reported as weak (w, <20% of tallest signal), medium (m, 21%–70% of tallest signal) or strong (s, >71% of tallest signal). Low and high resolution mass spectrometry was performed using the indicated techniques on either LCT Premier XE or TQD instruments (Waters, Centennial Court, Elstree, Hertfordshire, UK) equipped with Acquity UPLC and a lock-mass electrospray ion source. For accurate mass measurements the deviation from the calculated formula is reported in ppm. Melting points were recorded on an Optimelt automated melting point system (Lambda Photometrics, Lambda House, Hertfordshire, UK) with a heating rate of 1 °C/min and are uncorrected. The flow reactors systems used in this investigation were the manual control R series: R2+ with R4 heater unit (RS-100 system) available from Vapourtec Ltd. (https://www.vapourtec.com/) and the Polar Bear Plus system commercially available from Cambridge Reactor Design Ltd. (http://www.cambridgereactordesign.com/). The Polar Bear Plus flow reactor unit was modified by our workshop to house a removable copper plate which could be affixed to the top of the unit and housed two Uniqsis mixing chips [51].

### 3.2. Reactor Configuration for the Synthesis of Hydrazine Derivatives from Diazonium Salts ***1**–**18***

Three stock solutions were prepared and connected to the flow reactor feed lines for Pumps A–C (see Figure 3 for a pictorial layout). Pump A delivered hydrochloric acid (0.84 M), Pump B a solution of aqueous sodium nitrite (0.98 M) and pump C delivered an aqueous solution of ascorbic acid (0.35 M). In addition a further pump was used to provide the solutions of aniline as their HCl salts dissolved in water (0.35 M). The entire reactor was maintained under positive internal pressure using a 75 psi back pressure regulator at the exit of the reactor. To initiate the reaction each flow channel was pumped at 0.5 mL/min.

Progressing through the reactor; Channel A and B were mixed in a Uniqsis mixer chip of 0.27 mL internal volume (16 s residence time) before passing into a 2.75 mL PFA tubular residence coil (165 s residence time). The combined flow was then further united with the aniline solution mixing in a second Uniqsis mixer chip of 2 mL internal volume (80 s residence time). The reacting solution then passed into a 5 mL residence PFA foil coil (150 s residence time, the solution turns pale yellow to orange). The whole initial stage mixing unit was temperature regulated (10 °C) using a Polar Bear Plus flow reactor unit.

In the second stage reactor the freshly prepared diazonium mixture (combined flow rate 2 mL/min) was united at an Upchurch peek T-mixer with a solution of ascorbic acid delivered from Pump C set at 0.5 mL/min. The flow stream was then progressed into a 52 mL PFA flow coil (20.8 min residence time).

The isolation of certain intermediate hydroxamic esters, namely **13**–**18**, could be achieved by immediate extraction of the reactor output with ethyl acetate (5 volumes) and washing the organic phase with sodium hydrogen carbonate (2 M; 2×). The organic solution was dried over MgSO_4_, filtered and concentrated in vacuo to provide a pale yellow solid which was triturated with a 1:1 mixture of hexane and acetone to furnishing the desired product.

Alternatively, the output solution was collected and left to stir for 1 h to ensure complete hydrolysis to the corresponding oxamic acid, **1**–**12**, had occurred. The products were isolated by basification of the reaction mixture pH ~ 9 followed by extraction with EtOAc (3 volumes). The aqueous solution was then acidified to pH ~ 4 and extracted with EtOAc (3 volumes), the organic phase was dried over MgSO_4_, the solvent evaporated to yield compounds **1**–**12**. Note: for compounds **1**–**12** a proton signal sites under the residual DMSO signal.

### 3.3. Product Characterization

*2-(2-(2-Bromophenyl)hydrazinyl)-2-oxoacetic acid* (**1**): 10 mmol scale, 2.05 g, 79%. ^1^H-NMR (400 MHz, DMSO-*d*_6_) δ 10.78 (br. s, 1H), 8.25 (s, 1H), 7.51–7.35 (m, 2H), 7.22 (td, *J* = 7.7, 1.4 Hz, 1H), 6.78–6.63 (m, 2H). ^13^C-NMR (101 MHz, DMSO-*d*_6_) δ 162.0 (C), 159.1 (C), 145.3 (C), 132.9 (CH), 128.8 (CH), 120.9 (CH), 113.6 (CH), 107.5 (C). FT-IR ν_max_ 3329 (w), 3230 (w), 2900 (w), 1729 (m), 1670 (s), 1593 (m), 1451 (m), 1215 (s), 747 (s), 634 (m) cm^−1^. LC-MS (ESI) R_t_ = 1.98 min, *m*/*z* 258.9 (M + H), HR-MS (ES+) calculated for C_8_H_7_BrN_2_O_3_ 258.9718, found 258.9729 (Δ = 4.2 ppm).

*2-(2-(3-Bromophenyl)hydrazinyl)-2-oxoacetic acid* (**2**): 10 mmol scale, 2.07 g, 80%. ^1^H-NMR (400 MHz, DMSO-*d*_6_) δ 10.70 (br. s, 1H), 8.22 (s, 1H), 7.11 (t, *J* = 8.0 Hz, 1H), 6.89 (m, 1H), 6.82 (m, 1H), 6.71 (ddd, *J* = 8.2, 2.2, 0.9 Hz, 1H). ^13^C-NMR (101 MHz, DMSO-*d*_6_) δ 162.1 (C), 159.1 (C), 150.6 (C), 131.2 (CH), 122.5 (C), 121.7 (CH), 114.9 (CH), 111.8 (CH). FT-IR ν_max_ 3339 (w), 3207 (w), 1730 (m), 1663 (s), 1594 (s), 1219 (s), 1036 (m), 867 (m), 748 (s), 486 (m) cm^−1^. LC-MS (ESI) R_t_ = 1.74 min, *m*/*z* 258.9 (M + H), HR-MS (ES+) calculated for C_8_H_7_BrN_2_O_3_ 258.9718, found 258.9728 (Δ = 3.9 ppm).

*2-(2-(4-Bromophenyl)hydrazinyl)-2-oxoacetic acid* (**3**): 20 mmol scale, 4.86 g, 94%. ^1^H-NMR (400 MHz, DMSO-*d*_6_) δ 10.68 (br. s, 1H), 8.12 (br. s, 1H), 7.31 (d, *J* = 8.7 Hz, 2H), 6.72–6.62 (d, *J* = 8.7 Hz, 2H). ^13^C-NMR (101 MHz, MeOD-*d*_4_) δ 160.7 (C), 158.7 (C), 147.2 (C), 131.4 (2 × CH), 114.6 (2 × CH), 111.52 (C). FT-IR ν_max_ 3295 (m), 3028 (w), 1730 (m), 1700 (s), 1482 (s), 1399 (m), 1199 (s), 1176 (m), 824 (s), 704 (m), 477 (s) cm^−1^. LC-MS (ESI) R_t_ = 1.77 min, *m*/*z* 258.97 (M + H), HR-MS (ES+) calculated for C_8_H_7_BrN_2_O_3_ 258.9718, found 258.9719 (Δ = 0.4 ppm).

*2-(2-(2-Chlorophenyl)hydrazinyl)-2-oxoacetic acid* (**4**): 20 mmol scale, 2.91 g, 68%. ^1^H-NMR (400 MHz, DMSO-*d*_6_) δ 10.74 (s, 1H), 7.64 (s, 1H), 7.30 (d, *J* = 7.8 Hz, 1H), 7.17 (t, *J* = 7.7 Hz, 1H), 6.78 (d, *J* = 7.6 Hz, 1H), 6.75 (d, *J* = 7.6 Hz, 1H). ^13^C-NMR (101 MHz, DMSO-*d*_6_) δ 162.1 (C), 159.2 (C), 144.3 (C), 129.7 (C), 128.2 (CH), 120.3 (CH), 117.8 (CH), 113.4 (CH). FT-IR ν_max_ 3339 (w), 3207 (w), 1730 (m), 1663 (s), 1490 (m), 1257 (m), 1220 (s), 1037 (m), 748 (s), 486 (s) cm^−1^. LC-MS (ESI) R_t_ = 1.69 min, *m*/*z* 215.0 (M + H), HR-MS (ES+) calculated for C_8_H_7_ClN_2_O_3_ 215.0223, found 215.0231 (Δ = 3.7 ppm).

*2-(2-(3-Chlorophenyl)hydrazinyl)-2-oxoacetic acid* (**5**): 10 mmol scale, 1.78 g, 83%. ^1^H-NMR (400 MHz, DMSO-*d*_6_) δ 10.71 (br. s, 1H), 8.23 (br. s, 1H), 7.17 (t, *J* = 8.0 Hz, 1H), 6.81–6.61 (m, 3H). ^13^C-NMR (101 MHz, DMSO-*d*_6_) δ 162.1 (C), 159.1 (C), 150.4 (C), 134.0 (C), 130.9 (CH), 118.8 (CH), 112.0 (CH), 111.4 (CH). FT-IR ν_max_ 3351 (w), 3291 (w), 1747 (s), 1674 (m), 1560 (s), 1473 (m), 1249 (m), 1206 (s), 792 (m), 479 (s) cm^−1^. LC-MS (ESI) R_t_ = 1.72 min, *m*/*z* 215.0 (M + H), HR-MS (ES+) calculated for C_8_H_7_ClN_2_O_3_ 215.0223, found 215.0224 (Δ = 0.5 ppm).

*2-(2-(4-Chlorophenyl)hydrazinyl)-2-oxoacetic acid* (**6**): 10 mmol scale, 1.93 g, 90%. ^1^H- NMR (400 MHz, DMSO-*d*_6_) δ 10.69 (br. s, 1H), 8.11 (br. s, 1H), 7.20 (d, *J* = 9.0 Hz, 2H), 6.92 (d, *J* = 9.0 Hz, 2H). ^13^C-NMR (101 MHz, DMSO-*d*_6_) δ 162.1 (C), 159.0 (C), 147.8 (C), 129.0 (2CH), 122.7 (C), 114.3 (2 CH). FT-IR ν_max_ 3288 (w), 2991 (w), 1735 (w), 1696 (s), 1596 (w), 1489 (s), 1202 (w), 1175 (m), 827 (s), 481 (s) cm^−1^. LC-MS (ESI) R_t_ = 1.75 min, *m*/*z* 215.0 (M + H), HR-MS (ES+) calculated for C_8_H_7_ClN_2_O_3_ 215.0223, found 215.0230 (Δ = 3.3 ppm).

*2-(2-(2-Nitrophenyl)hydrazinyl)-2-oxoacetic acid* (**7**): 30 mmol scale, 6.01 g, 89%. ^1^H-NMR (400 MHz, DMSO-*d*_6_) δ 11.08 (br. s, 1H), 9.36 (br. s, 1H), 8.11 (dd, *J* = 8.5, 1.5 Hz, 1H), 7.60 (ddd, *J* = 8.5, 6.9, 1.5 Hz, 1H), 7.09 (dd, *J* = 8.6, 1.2 Hz, 1H), 6.90 (ddd, *J* = 8.4, 7.0, 1.3 Hz, 1H). ^13^C-NMR (101 MHz, DMSO-*d*_6_) δ 161.6 (C), 158.6 (C), 144.8 (C), 136.9 (CH), 132.3 (C), 126.3 (CH), 118.6 (CH), 115.2 (CH). FT-IR ν_max_ 3228 (w), 1757 (m), 1672 (m), 1612 (m), 1319 (s), 1261 (s), 1153 (s), 951 (m), 737 (s), 490(s) cm^−1^. LC-MS (ESI) R_t_ = 1.44 min, *m*/*z* 226.1 (M + H), HR-MS (ES+) calculated for C_8_H_7_N_3_O_5_ 226.0464, found 226.0473 (Δ = 4.0 ppm).

*2-(2-(3-Nitrophenyl)hydrazinyl)-2-oxoacetic acid* (**8**): 28 mmol scale, 5.80 g, 92%. ^1^H-NMR (400 MHz, DMSO-*d*_6_) δ 10.88 (s, 1H), 8.61 (s, 1H), 7.58 (d, *J* = 8.1 Hz, 1H), 7.48 (d, *J* = 6.8 Hz, 1H), 7.44 (d, *J* = 8.1 Hz, 1H), 7.15 (d, *J* = 6.9 Hz, 1H). ^13^C-NMR (101 MHz, DMSO-*d*_6_) δ 162.0 (C), 159.0 (C), 150.1 (C), 149.0 (C), 130.7 (CH), 119.0 (CH), 113.7 (CH), 106.2 (CH). FT-IR ν_max_ 3361 (w), 3270 (w), 1757 (m), 1664 (m), 1526 (s), 1489 (m), 1349 (s), 1205 (m), 732 (s), 494 (s) cm^−1^. LC-MS (ESI) R_t_ = 1.34 min, *m*/*z* 226.1 (M + H), HR-MS (ES+) calculated for C_8_H_7_N_3_O_5_ 226.0464, found 226.0469 (Δ = 2.2 ppm).

*2-(2-(4-Nitrophenyl)hydrazinyl)-2-oxoacetic acid* (**9**): 12 mmol scale, 2.43 g, 90%. ^1^H-NMR (400 MHz, DMSO-*d*_6_) δ 10.97 (s, 1H), 9.22 (s, 1H), 8.08 (d, *J* = 9.1 Hz, 2H), 6.78 (d, *J* = 9.1 Hz, 2H). ^13^C-NMR (101 MHz, DMSO-*d*_6_) δ 161.7 (C), 158.8 (C), 154.4 (C), 138.8 (2 CH), 126.3 (C), 111.3 (2 CH). FT-IR ν_max_ 3300 (w), 1699 (m), 1592 (s), 1499 (s), 1526 (s), 1309 (s), 1215 (m), 1109 (s), 732 (s), 840 (m) cm^−1^. LC-MS (ESI) R_t_ = 1.29 min, *m*/*z* 226.1 (M + H), HR-MS (ES+) calculated for C_8_H_7_N_3_O_5_ 226.0464, found 226.0470 (Δ = 2.7 ppm).

*2-(2-(2-Methoxyphenyl)hydrazinyl)-2-oxoacetic acid* (**10**): 30 mmol scale, 4.22 g, 67%. ^1^H-NMR (400 MHz, DMSO-*d*_6_) δ 10.69 (br. s, 1H), 7.11 (br. m, 1H), 6.78 (m, 4H), 3.81 (s, 3H). ^13^C-NMR (101 MHz, DMSO) δ 162.2 (C), 158.7 (C), 146.9 (C), 137.6 (C), 121.1 (CH), 119.8 (CH), 112.0 (CH), 110.9 (CH), 55.9 (CH_3_). FT-IR ν_max_ 3325 (w), 3209 (w), 3049 (w), 1709 (m), 1679 (s), 1499 (s), 1346 (s), 1211 (s), 1028 (m), 733 (s) cm^−1^. LC-MS (ESI) R_t_ = 1.70 min, *m*/*z* 211.1 (M + H), HR-MS (ES+) calculated for C_9_H_10_N_2_O_4_ 211.0719, found 211.0719 (Δ = 0 ppm).

*2-Oxo-2-(2-(m-tolyl)hydrazinyl)acetic acid* (**11**): 30 mmol scale, 4.19 g, 72%. ^1^H-NMR (400 MHz, DMSO-*d*_6_) δ 10.63 (br. s, 1H), 7.93 (br. s, 1H), 7.06 (m, 1H), 6.57 (m, 3H), 2.23 (s, 3H). ^13^C-NMR (101 MHz, DMSO-*d*_6_) δ 162.3 (C), 159.1 (C), 148.8 (C), 138.3 (C), 129.1 (CH), 120.2 (CH), 113.3 (CH), 110.1 (CH), 21.7 (CH_3_). FT-IR ν_max_ 3283 (m), 2916 (w), 3049 (w), 1758 (m), 1683 (s), 1612 (m), 1346 (m), 1169 (s), 1956 (m), 689 (s) cm^−1^. LC-MS (ESI) R_t_ = 1.48 min, *m*/*z* 195.1 (M + H), HR-MS (ES+) calculated for C_9_H_10_N_2_O_3_ 195.0770, found 195.0773 (Δ = 1.5 ppm).

*2-Oxo-2-(2-(p-tolyl)hydrazinyl)acetic acid* (**12**): 25 mmol scale, 3.73 g, 77%. ^1^H-NMR (400 MHz, DMSO-*d*_6_) δ 10.61 (br. s, 1H), 7.80 (br. s, 1H), 6.97 (d, *J* = 8.2 Hz, 2H), 6.64 (d, *J* = 8.2 Hz, 2H), 2.18 (s, 3H). ^13^C-NMR (101 MHz, DMSO-*d*_6_) δ 162.4 (C), 159.0 (C), 146.5 (C), 129.6 (2CH), 128.0 (C), 113.1 (2 × CH), 20.6 (CH_3_). FT-IR ν_max_ 3326 (w), 3177 (w), 1755 (m), 1683 (s), 1511 (m), 11354 (s), 1280 (m), 940 (m), 807(s), 477(s) cm^−1^. LC-MS (ESI) R_t_ = 1.82 min, *m*/*z* 195.08 (M + H), HR-MS (ES+) calculated for C_9_H_11_N_2_O_3_ 195.0770, found 195.0775 (Δ = 2.6 ppm).

*(3R,4S)-4-Hydroxy-2-oxotetrahydrofuran-3-yl-2-(2-(4-bromophenyl)hydrazinyl)-2-oxoacetate* (**13**): 5 mmol scale, 687 mg, 53%. ^1^H-NMR (700 MHz, DMSO-*d*_6_) δ 11.00 (d, *J* = 2.5 Hz, 1H), 8.22 (d, *J* = 2.7 Hz, 1H), 7.30 (d, *J* = 7.2 Hz, 2H), 6.67 (d, *J* = 7.2 Hz, 2H), 6.15 (d, *J* = 4.7 Hz, 1H), 5.66 (d, *J* = 7.9 Hz, 1H), 4.67 (qd, *J* = 7.9, 4.7 Hz, 1H), 4.49 (dd, *J* = 8.5, 7.5 Hz, 1H), 4.04 (t, *J* = 8.3 Hz, 1H). ^13^C-NMR (176 MHz, DMSO-*d*_6_) δ 170.6 (C), 159.1 (C), 156.3 (C), 147.8 (C), 131.9 (2 × CH), 114.8 (2 × CH), 110.5 (C), 76.4 (CH), 70.0 (CH), 69.8 (CH_2_). ^1^H-NMR (700 MHz, methanol-*d*_4_) δ 7.29 (d, *J* = 7.4 Hz, 2H), 6.74 (d, *J* = 7.4 Hz, 2H), 4.40 (dd, *J* = 9.0, 7.0 Hz, 1H), 4.27 (q, *J* = 7.0 Hz, 1H), 4.17 (d, *J* = 7.0 Hz, 1H), 3.92 (dd, *J* = 9.0, 7.0 Hz, 1H). ^13^C-NMR (176 MHz, methanol-*d*_4_) δ 175.9 (C), 160.0 (C), 157.6 (C), 147.1 (C), 131.4 (2 × CH), 114.6 (2 × CH), 111.6 (C), 73.36 (CH), 72.68 (CH), 69.80 (CH_2_). FT-IR ν_max_ 3457 (w), 3355 (w), 3224 (w), 1782 (m), 1759 (s), 1690 (m), 1478 (m), 1204 (m), 812 (s), 504 (s) cm^−1^. LC-MS (ESI) 1.91 R_t_ *m*/*z* 359.3 and 719.3. LC-MS (ESI) R_t_ = 2.56 min, *m*/*z* 359 (M + H), HR-MS (ES+) calculated for C_12_H_11_BrN_2_O_6_ 358.9879, found 358.9891 (Δ = 3.3 ppm). X-ray CCDC 1485241; P2ac2ab; a = 5.3554(3), b = 8.6070(5), c = 29.1832(16); α = 90°, β = 90°, γ = 90°.

*(3R,4S)-4-Hydroxy-2-oxotetrahydrofuran-3-yl-2-(2-(4-chlorophenyl)hydrazinyl)-2-oxoacet*ate (**14**): 5 mmol scale, 644 mg, 60%. ^1^H-NMR (700 MHz, DMSO-*d*_6_) δ 11.00 (d, *J* = 2.6 Hz, 1H), 8.21 (d, *J* = 2.8 Hz, 1H), 7.19 (d, *J* = 7.2 Hz, 2H), 6.72 (d, *J* = 7.2 Hz, 2H), 6.15 (d, *J* = 4.9 Hz, 1H), 5.66 (d, *J* = 7.9 Hz, 1H), 4.66 (td, *J* = 7.7, 4.8 Hz, 1H), 4.49 (dd, *J* = 8.5, 7.7 Hz, 1H), 4.04 (t, *J* = 8.5 Hz, 1H). ^13^C-NMR (176 MHz, DMSO-*d*_6_) δ 170.6 (C), 159.2 (C), 156.3 (C), 147.4 (C), 129.1 (2CH), 122.9 (C), 114.3 (2CH), 76.4 (CH), 69.9 (CH_2_), 69.8 (CH). ^1^H-NMR (400 MHz, methanol-*d*_4_) δ 7.20 (d, *J* = 7.3 Hz, 2H), 6.85 (d, *J* = 7.3 Hz, 2H), 5.71 (d, *J* = 7.7 Hz, 1H), 4.78 (q, *J* = 7.7 Hz, 1H), 4.58 (dd, *J* = 8.9, 7.7 Hz, 1H), 4.13 (dd, *J* = 8.9, 8.0 Hz, 1H). ^13^C-NMR (176 MHz, methanol-*d*_4_) δ 170.6 (C), 159.2 (C), 156.3 (C), 147.4 (C), 129.1 (2CH), 122.9 (C), 114.3 (2CH), 76.4 (CH), 69.9 (CH), 69.8 (CH_2_). FT-IR ν_max_ 3448 (w), 3362 (w), 3227 (w), 1785 (m), 1761 (s), 1692 (m),1508 (m), 1029 (s), 821 (m), 506 (s) cm^−1^. LC-MS (ESI) R_t_ = 2.35 min, *m*/*z* 315.0 (M + H), HR-MS (ES+) calculated for C_12_H_11_ClN_2_O_6_ 314.0384, found 314.0386 (Δ = 0.6 ppm). X-ray CCDC 1485245; P2ac2ab; a = 5.3548(3), b = 8.5893(5), c = 28.7810(16); α = 90°, β = 90°, γ = 90°.

*(3R,4S)-4-Hydroxy-2-oxotetrahydrofuran-3-yl-2-(2-(2-nitrophenyl)hydrazinyl)-2-oxoacetate* (**15**): 10 mmol scale, 878 mg, 78%. ^1^H-NMR (400 MHz, DMSO-*d*_6_) δ 11.41 (br. s, 1H), 9.46 (br. s, 1H), 8.13 (dd, *J* = 8.6, 1.6 Hz, 1H), 7.63 (ddd, *J* = 8.6, 7.0, 1.6 Hz, 1H), 7.12 (d, *J* = 8.6 Hz, 1H), 6.93 (ddd, *J* = 8.6, 7.0, 1.6 Hz, 1H), 6.20 (d, *J* = 5.0 Hz, 1H), 5.73 (d, *J* = 7.7 Hz, 1H), 4.72 (qd, *J* = 7.7, 3.4 Hz, 1H), 4.53 (dd, *J* = 8.5, 7.7 Hz, 1H), 4.09 (t, *J* = 8.5 Hz, 1H). ^13^C-NMR (101 MHz, DMSO-*d*_6_) δ 170.6 (C), 158.8 (C), 155.9 (C), 144.4 (C), 136.9 (C), 132.5 (CH), 126.4 (CH), 118.9 (CH), 115.1 (CH), 76.5 (CH), 70.2 (CH_2_), 69.8 (CH). FT-IR ν_max_ 3366 (w), 3321 (w), 3271 (w), 1790 (s), 1721 (s), 1701 (s), 1611 (m), 1494 (s), 1350 (m), 1153 (s), 752 (s) cm^−1^. LC-MS (ESI) R_t_ = 2.06 min, *m*/*z* 326.1 (M + H), HR-MS (ES+) calculated for C_12_H_12_N_3_O_8_ 326.0624, found 326.0629 (Δ = 1.5 ppm).

*(3R,4S)-4-Hydroxy-2-oxotetrahydrofuran-3-yl-2-(2-(2-methoxyphenyl)hydrazinyl)-2-oxoacetate* (**16**): 5 mmol scale, 441 mg, 42%. ^1^HNMR (400 MHz, DMSO-*d*_6_) δ 11.02 (br. s, 1H), 7.33 (br. s, 1H), 6.92 (dd, *J* = 7.7, 1.7 Hz, 1H), 6.85–6.73 (m, 2H), 6.66 (dd, *J* = 7.5, 1.9 Hz, 1H), 6.21 (d, *J* = 4.9 Hz, 1H), 5.70 (d, *J* = 8.0 Hz, 1H), 4.71 (qd, *J* = 8.0, 4.9 Hz, 1H), 4.52 (dd, *J* = 8.5, 7.5 Hz, 1H), 4.07 (t, *J* = 8.5 Hz, 1H), 3.83 (s, 3H). ^13^CNMR (101 MHz, DMSO-*d*_6_) δ 170.7 (C), 159.2 (C), 156.1 (C), 147.0 (C), 137.2 (C), 121.1 (CH), 112.0 (CH), 111.9 (CH), 111.1 (CH), 76.4 (CH), 70.0 (CH_2_), 69.8 (CH), 55.9 (CH_3_). FT-IR ν_max_ 3432 (w), 3363 (w), 3225 (w), 1784 (m), 1761 (s), 1702 (m), 1499 (s), 1133 (m), 1019 (s), 734 (s), 493 (s) cm^−1^. LC-MS (ESI) R_t_ = 2.03 min, *m*/*z* 311.1 (M + H), HR-MS (ES+) calculated for C_13_H_15_N_2_O_7_ 311.0879, found 311.0879 (Δ = 0.0 ppm).

*(3R,4S)-4-hydroxy-2-oxotetrahydrofuran-3-yl-2-(2-(4-methoxyphenyl)hydrazinyl)-2-oxoacetate* (**17**): 5 mmol scale, 525 mg, 50%. ^1^H-NMR (400 MHz, DMSO-*d*_6_) δ 10.98 (br. s, 1H), 7.75 (br. s, 1H), 6.80 (d, *J* = 8.9 Hz, 2H), 6.72 (d, *J* = 8.9 Hz, 2H), 6.19 (d, *J* = 4.9 Hz, 1H), 5.69 (d, *J* = 7.9 Hz, 1H), 4.70 (qd, *J* = 7.6, 4.9 Hz, 1H), 4.52 (dd, *J* = 8.5, 7.6 Hz, 1H), 4.07 (t, *J* = 8.5 Hz, 1H), 3.68 (s, 3H). ^13^C-NMR (101 MHz, DMSO-*d*_6_) δ 170.7 (C), 159.4 (C), 156.3 (C), 153.6 (C), 142.2 (C), 115.5 (2 × CH), 114.8 (2 × CH), 76.3 (CH), 70.0 (CH_2_), 69.8 (CH), 55.7 (CH_3_). FT-IR ν_max_ 3445 (w), 3363 (w), 3224 (w), 1785 (m), 1760 (s), 1700 (m), 1508 (s), 1122 (m), 1030 (s), 820 (s), 493 (s) cm^−1^. LC-MS (ESI) R_t_ = 1.81 min, *m*/*z* 311.09 (M + H), HR-MS (ES+) calculated for C_13_H_15_N_2_O_7_ 311.0879, found 311.0891 (Δ = 3.9 ppm).

*(3R,4S)-4-Hydroxy-2-oxotetrahydrofuran-3-yl 2-oxo-2-(2-(p-tolyl)hydrazinyl)acetate* (**18**): 5 mmol scale, 602 mg, 62%. ^1^H-NMR (400 MHz, DMSO-*d*_6_) δ 10.96 (br. s, 1H), 7.89 (br. s, 1H), 6.99 (d, *J* = 8.2 Hz, 2H), 6.66 (d, *J* = 8.2 Hz, 2H), 6.19 (d, *J* = 4.9 Hz, 1H), 5.69 (d, *J* = 7.9 Hz, 1H), 4.75–4.64 (m, 1H), 4.52 (t, *J* = 8.2 Hz, 1H), 4.07 (t, *J* = 8.2 Hz, 1H), 2.19 (s, 3H). ^13^C-NMR (101 MHz, DMSO-*d*_6_) δ 170.7 (C), 159.3 (C), 156.3 (C), 146.1 (C), 129.7 (2CH), 128.4 (C), 113.1 (2CH), 76.3 (CH), 70.0 (CH_2_), 69.8 (CH), 20.6 (CH_3_). FT-IR ν_max_ 3429 (w), 3310 (w), 3260 (w), 1765 (s), 1511 (m), 1193 (s), 1014 (m), 805 (s), 643 (m), 506 (s) cm^−1^. LC-MS (ESI) R_t_ = 2.17 min, *m*/*z* 295.09 (M + H), HR-MS (ES+) calculated for C_13_H_15_N_2_O_7_ 295.0930, found 295.0928 (Δ = 1.7 ppm).

*4-[[(1, 1’-Dimethylethoxy)carbonyl]amino]benzenediazonium tetrafluoroborate* (**19**) [79]: Stock solutions of isobutyl nitrite (0.345 M) trimethylsilyl chloride (0.345 M) and *tert*-butyl-4-aminophenylcarbamate (0.30 M) in 2:1 solvent mixture of dichloroethane and MeCN were prepared. At flow rates of 0.25 mL/min the solutions of trimethylsilyl chloride and isobutyl nitrite were pumped and united at a Upchurch PEEK T-mixer then immediately combined at a second T-mixer the solution of aniline. The combined flow stream was progressed into a PFA tubular reactor (internal volume 20 mL, residence time 26.7 min) maintained at 20 °C. Samples of the reactor output were taken every 10 min for direct ^1^H-NMR analysis to assess conversion (which was consistent at 98% after the first 15 min). The bulk reactor output was collected (for 2 h) into a stirred round bottom flask containing a suspension of sodium tetrafluoroborate (1.087 g, 1.1 equiv.) in EtOH (20 mL). The suspension was evaporated and triturated with a 3:1 mixture of diethyl ether and MeCN to give a pale off white solid 2.266 g, 82%. ^1^H-NMR (400 MHz, DMSO-*d*_6_) δ/ppm: 10.97 (s, 1H), 8.58 (d, *J* = 8.0 Hz, 2Hz), 7.95 (d, *J* = 8.0 Hz, 2H), 1.51 (s, 9 H). ^13^C-NMR (101 MHz, DMSO-*d*_6_) δ/ppm: 152.3 (C), 151.5 (C), 135.5 (2 × CH), 119.2 (2 × CH), 103.5 (C), 82.2 (C), 28.2 (3 × CH_3_). ^19^F-NMR (376 MHz, DMSO-*d*_6_) δ/ppm: −148.3 (s). IR (neat) ν 3308 (w), 2975 (w), 2246 (m), 1741 (m), 1579 (s), 1527 (s), 1433 (m), 1232 (s), 1154 (s), 1090 (s), 1056 (s), 1006 (s), 839 (s), 519 (s) cm^−1^. LC-MS (ESI) 220.2 (M+). HR-MS (ESI) calculated for C_11_H_14_N_3_O_2_ 220.1086, found 220.1099 (Δ = 5.9 ppm).

*(E)-1-((3-Fluorophenyl)diazenyl)naphthalene-2-ol* (**20**): Assessed loading 14.2 mmol, 89%; Isolated yield 3.44 g, 81%. ^1^H-NMR (400 MHz, CDCl_3_) δ/ppm: 16.07 (1H, s), 8.53–8.45 (1H, m), 7.70 (1H, d, *J* = 9.5 Hz), 7.62–7.52 (2H, m), 7.52–7.46 (1H, m), 7.45–7.33 (3H, m), 7.02–6.91 (1H, m), 6.81 (1H, d, *J* = 9.5 Hz). ^13^C-NMR (101 MHz, CDCl_3_) δ/ppm: 173.5 (C), 163.8 (CF, d, *J* = 247 Hz), 146.2, (C, d, *J* = 9 Hz), 141.0 (CH), 133.4 (C), 130.8 (CH, d, *J* = 9 Hz), 130.4 (C), 129.1 (CH), 128.7 (CH), 128.2 (C), 126.2 (CH), 125.0 (CH), 121.9 (CH), 114.7 (CH, d, *J* = 3 Hz), 113.7 (CH, d, *J* = 22 Hz), 104.7 (CH, d, *J* = 26 Hz). ^19^F-NMR (376 MHz, CDCl_3_) δ/ppm: −110.87 (s). IR (neat) ν 1611.1 (m), 1596.0 (m), 1495.7 (m), 1251.2 (m), 1213.2 (m), 1109.8 (m), 987.9 (m), 865.8 (m), 834.3 (s), 768.9 (s), 746.0 (s), 675.1 (s), 514.0 (s) 454.6 (m) cm^−1^. LC-MS (ESI) 267.1 (M + H). HR-MS (ESI) calculated for C_16_H_12_N_2_OF 267.0934, found 267.0928 (Δ = −2.2 ppm). Melting range: 136.9–139.7 °C.

*(E)-1-((3-(Trifluoromethyl)phenyl)diazenyl)naphthalene-2-ol* (**21**) [80]: Assessed loading 12.7 mmol, 86%; Isolated 3.45 g, 74%. ^1^H-NMR (400 MHz, CDCl_3_) δ/ppm: 16.07 (1H, s), 8.48 (1H, d, *J* = 7.6 Hz), 7.94 (1H, s), 7.81 (1H, d, *J* = 8.0 Hz), 7.70 (1H, d, *J* = 9.5 Hz), 7.63–7.53 (3H, m), 7.51 (1H, d, *J* = 7.6 Hz), 7.40 (1H, ddd, *J* = 8.0, 7.2, 1.2 Hz), 6.81 (1H, d, *J* = 9.5 Hz). ^13^C-NMR (101 MHz, CDCl_3_) δ/ppm: 173.6 (C), 145.0 (C), 141.2 (CH), 133.3 (C), 132.2 (C, q, *J* = 33 Hz), 130.6 (C), 130.1 (CH), 129.2 (CH), 128.8 (CH), 128.3 (C), 126.4 (CH), 125.0 (CH), 123.8 (CF_3_, q, *J* = 274 Hz), 123.2 (CH, q, *J* = 4 Hz), 121.9 (CH), 121.3 (CH), 114.8 (CH, q, *J* = 4 Hz). ^19^F-NMR (376 MHz, CDCl_3_) δ/ppm: -62.83 (s). IR (neat) ν 1739.4 (m), 1617.1 (m), 1496.7 (m), 1448.5 (m), 1313.7 (m), 1246.8 (s), 1203.7 (s), 1116.7 (s), 833.9 (s), 795.2 (s), 754.9 (s), 690.4 (s), 663.2 (s), 504.8 (s) cm^−1^. LC-MS (ESI) 317.5 (M + H). HR-MS (ESI) calculated for C_17_H_12_N_2_OF_3_ 317.0902, found 317.0901 (Δ = −0.3 ppm). Melting range: 164.8–167.5 °C.

*(E)-4-((2-Hydroxynaphthalen-1-yl)diazenyl)benzonitrile* (**22**) [81,82]: Assessed loading 9.3 mmol, 93%; Isolated 2.27 g, 83%.^1^H-NMR (400 MHz, CDCl_3_) δ/ppm 16.10 (1H, s), 8.40 (1H, d, *J* = 8.0 Hz), 7.71 (2H, d, *J* = 9.0 Hz), 7.66 (2H, d, *J* = 9.0 Hz), 7.65–7.75 (2H, m), 7.53 (1H, m), 7.42 (1H, m), 6.71 (1H, d, *J* = 9.6 Hz). ^13^C-NMR (101 MHz, CDCl_3_) δ/ppm: 179.1 (C), 146.5 (C), 143.0 (CH), 133.8 (2CH), 133.1 (C), 131.5 (C), 129.7 (CH), 129.1 (CH), 128.5 (C), 127.3 (CH), 126.1 (CH), 122.3 (CH), 118.8 (C), 117.3 (2CH), 108.4 (C). IR (neat) ν 3066.9 (w), 2219.6 (m), 1738.7 (w), 1604.2 (m), 1496.0 (s), 1450.0 (s), 1395.8 (s), 1253.8 (s), 1205.6 (s), 1148.4 (s), 1093.4 (m), 983.1 (m), 837.0 (s), 757.6 (s), 513.5 (s) cm^−1^. LC-MS (ESI) 274.0 (M + H). HR-MS (ESI) calculated for C_17_H_12_N_3_O 274.0980, found 274.0980 (Δ = 0.0 ppm). Melting range: 141.6–143.3 °C.

*(E)-1-((3-Methoxyphenyl)diazenyl)naphthalene-2-ol* (**23**) [83,84]: Assessed loading 21.3 mmol, 91%; Isolated 5.72 g, 88%. ^1^H-NMR (400 MHz, DMSO-*d*_6_) δ/ppm: 15.76 (s, 1H), 8.52 (dd, *J* = 1.1, 8.3 Hz, 1H), 7.95 (d, *J* = 9.4 Hz, 1H), 7.78 (d, *J* = 8.3 Hz, 1H), 7.62 (ddd, *J* = 1.4, 7.1, 8.3 Hz, 1H), 7.50–7.40 (m, 4H), 6.95 (dt, *J* = 2.2, 7.1 Hz, 1H), 6.91 (d, *J* = 9.4 Hz, 1H), 3.87 (s, 3H). ^13^C-NMR (101 MHz, DMSO-*d*_6_) δ/ppm: 170.2 (C), 160.9 (C), 146.6 (C), 140.7 (CH), 133.2 (C), 131.1 (CH), 129.7 (C), 129.6 (CH), 129.4 (CH), 128.3 (C), 126.4 (CH), 124.6 (CH), 121.8 (CH), 114.5 (CH), 111.9 (CH), 104.0 (CH), 55.9 (CH_3_). IR (neat) ν 2966 (w), 2838 (w), 1602 (w), 1558 (m), 1487 (s), 1439 (m), 1247 (s), 1115 (s), 1041 (s), 987 (m), 863 (s), 839 (s), 766 (s), 755 (s), 676 (s), 517 (m) cm^−1^. LC-MS (ESI) 279.2 (M + H). HR-MS (ESI) calculated for C_17_H_15_N_2_O_2_ 279.1134, found 279.1137 (Δ = 1.1 ppm). Melting range: 138.7–140.9 °C.

*(E)-1-((4-Methoxyphenyl)diazenyl)naphthalene-2-ol* (**24**) [36,85]: Assessed loading 22.9 mmol, 89%; Isolated 5.80 g, 81%. ^1^H-NMR (400 MHz, CDCl_3_) δ/ppm: 15.71 (1H, s), 8.71 (1H, d, *J* = 8.4 Hz), 7.80 (2H, d, *J* = 9.0 Hz), 7.74 (1H, d, *J* = 9.2 Hz), 7.67 (1H, d, *J* = 7.6 Hz), 7.57 (1H, ddd, *J* = 8.4, 7.7, 1.2 Hz), 7.39 (1H, ddd, *J* = 8.4, 7.8, 1.2 Hz), 7.04 (1H, d, *J* = 9.0 Hz), 7.00 (2H, d, *J* = 9.1 Hz), 3.86 (3H, s). ^13^C-NMR (101 MHz, CDCl_3_) δ/ppm: 161.4 (C), 160.6 (C), 141.8 (C), 136.7 (CH), 133.3 (C), 129.5 (C), 128.3 (CH), 128.1 (C+CH), 124.8 (CH), 122.2 (CH), 122.0 (2CH), 121.6 (CH), 114.8 (2CH), 55.6 (CH_3_). IR (neat) ν 2836.6 (w), 1601.0 (m), 1502.1 (s), 1299.7 (m), 1158.5 (m), 1031.2 (m), 826.5 (m), 754.2 (m), 512.7 (m) cm^−1^. LC-MS (ESI) 279.1 (M + H). HR-MS (ESI) calculated for C_17_H_15_N_2_O_2_ 279.1134, found 279.1135 (Δ = 0.4 ppm). Melting range: 133.8–136.2 °C.

*(E)-3-Fluoro-4-((2-hydroxynaphthalen-1-yl)diazenyl)benzonitrile* (**25**): Assessed loading 11.6 mmol, 87%; Isolated 3.08 g, 79%. ^1^H-NMR (400 MHz, CDCl_3_) δ/ppm: 16.00 (1H, s), 8.35 (1H, d, *J* = 8.0 Hz), 8.06 (1H, t, *J* = 8.0 Hz), 7.66 (1H, d, *J* = 9.7 Hz), 7.50–7.60 (3H, m), 7.45–7.37 (m, 2H), 6.67 (1H, d, *J* = 9.7 Hz). ^13^C-NMR (101 MHz, CDCl_3_) δ/ppm: 180.4 (C), 151.2 (CF, d, *J* = 251 Hz), 143.6 (CH), 135.7 (C, d, *J* = 9 Hz), 132.8 (C), 132.7 (C), 129.8 (CH), 129.6 (CH, d, *J* = 4 Hz), 129.2 (CH), 128.7 (C), 127.8 (CH), 126.4 (CH), 122.6 (CH), 119.6 (CH, d, *J* = 21 Hz), 117.8 (C, d, *J* = 3 Hz), 117.0 (CH, d, *J* = 2 Hz), 107.7 (C, d, *J* = 9 Hz). ^19^F-NMR (376 MHz, CDCl_3_) δ/ppm: −128.16 (s). IR (neat) ν 2225.6 (m), 1613.5 (m), 1512.8 (m), 1452.2 (m), 1266.6 (m), 1202.9 (s), 1106.0 (m), 831.3 (s), 760.5 (s), 613.9 (m), 517.6 (s) cm^−1^. LC-MS (ESI) 292.1 (M + H). HR-MS (ESI) calculated for C_17_H_11_N_3_OF 292.0886, found 292.0894 (Δ = 2.7 ppm). Melting range: 227.4–229.2 °C.

*(E)-1-((2,4-Difluorophenyl)diazenyl)naphthalene-2-ol* (**26**): Assessed loading 11.0 mmol, 90%; Isolated 2.91 g, 84%. ^1^H-NMR (400 MHz, CDCl_3_) δ/ppm 15.77 (1H, s), 8.53 (1H, d, *J* = 8.4 Hz), 8.00 (1H, td, *J* = 8.9, 6.0 Hz), 7.72 (1H, d, *J* = 9.4 Hz), 7.61 (1H, m), 7.54 (1H, ddd, *J* = 8.3, 7.1, 1.3 Hz), 7.40 (1H, ddd, *J* = 8.0, 7.1, 1.2 Hz), 7.05–6.94 (2H, m), 6.91 (1H, d, *J* = 9.4 Hz). ^13^C-NMR (101 MHz, CDCl_3_) δ/ppm: 168.2 (C), 161.8 (CF, dd, *J* = 251, 11 Hz), 155.1 (CF, dd, *J* = 255, 12 Hz), 139.6 (CH), 133.2 (C), 131.2 (C, dd, *J* = 8, 4 Hz), 130.9 (C), 128.8 (CH), 128.6 (CH), 128.2 (C), 125.8 (CH), 123.7 (CH), 121.7 (CH), 118.1 (CH, dd, *J* = 10, 2 Hz), 112.4 (CH, dd, *J* = 23, 4 Hz), 104.7 (CH, dd, *J* = 27, 22 Hz). ^19^F-NMR (376 MHz, CDCl_3_) δ/ppm: −109.81 (d, *J* = 6 Hz), -123.56 (d, *J* = 6 Hz). IR (neat) ν 1620.2 (m), 1599.7 (m), 1507.3 (s), 1440.3 (m), 1271.6 (m), 1203.1 (s), 1132.8 (s), 957.6 (s), 834.0 (s), 801.2 (m), 747.6 (s), 718.9 (m) cm^−1^. LC-MS (ESI) 285.0 (M + H). HR-MS (ESI) calculated for C_16_H_11_N_2_OF_2_ 285.0839, found 285.0840 (Δ = 0.4 ppm). Melting range: 140.3–143.1 °C.

*(E)-1-((5-Chloro-2-phenoxyphenyl)diazenyl)naphthalene-2-ol* (**27**): Isolated 360 mg. ^1^H-NMR (400 MHz, CDCl_3_) δ/ppm: 8.51 (1H, d, *J* = 8.4 Hz), 8.08 (1H, d, *J* = 2.5 Hz), 7.64 (1H, d, *J* = 9.6 Hz), 7.60–7.50 (2H, m), 7.43–7.34 (3H, m), 7.20–7.07 (5H, m), 6.90 (1H, d, *J* = 8.7 Hz), 6.71 (1H, d, *J* = 9.6 Hz). ^13^C-NMR (101 MHz, CDCl_3_) δ/ppm 177.5 (C), 156.1 (C), 145.5 (C), 141.8 (CH), 135.7 (C), 133.4 (C), 131.4 (C), 130.0 (2CH), 129.9 (C), 129.3 (CH), 128.8 (CH), 128.2 (C), 126.6 (CH), 126.1 (CH), 126.0 (CH), 124.2 (CH), 122.1 (CH), 119.8 (CH), 118.8 (2CH), 116.5 (CH). IR (neat) ν 1617.3 (w), 1585.8 (w), 1473.9 (s), 1238.7 (m), 1197.8 (s), 1114.6 (m), 868.0 (m), 837.3 (s), 764.9 (s), 750.0 (s), 694.4 (s), 516.8 (m) cm^−1^. LC-MS (ESI) 375.0 (M + H). HR-MS (ESI) calculated for C_22_H_16_N_2_O_2_Cl 375.0900, found 375.0902 (Δ = 0.5 ppm). Melting range: 163.8-166.5 °C.

*(E)-1-((3-Chlorophenyl)diazenyl)naphthalene-2-ol* (**28**) [83,84]: Assessed loading 14.3 mmol, 86%; Isolated 3.89 g, 83%. ^1^H-NMR (400 MHz, CDCl_3_) δ/ppm: 16.02 (1H, s), 8.48 (1H, d, *J* = 8.0 Hz), 7.72 (1H, t, *J* = 2.0 Hz), 7.68 (1H, d, *J* = 9.5 Hz), 7.52–7.56 (2H, m), 7.48 (1H, ddd, *J* = 8.1, 2.0, 1.0 Hz), 7.39 (1H, ddd, *J* = 7.6, 6.6, 1.2 Hz), 7.35 (1H, d, *J* = 8.0 Hz), 7.22 (1H, ddd, *J* = 7.9, 2.0, 1.0 Hz), 6.80 (1H, d, *J* = 9.5 Hz). ^13^C-NMR (101 MHz, CDCl_3_) δ/ppm: 173.3 (C), 145.6 (C), 141.0 (CH), 135.6 (C), 133.3 (C), 130.5 (CH), 130.4 (C), 129.1 (CH), 128.7 (CH), 128.2 (C), 126.8 (CH), 126.2 (CH), 124.9 (CH), 121.9 (CH), 117.8 (CH), 116.9 (CH). IR (neat) ν 1617.2 (m), 1550.4 (m), 1499.3 (s), 1437.2 (m), 1252.3 (s), 1204.1 (s), 1068.8 (m), 862.3 (s), 833.9 (s), 779.9 (s), 752.5 (s), 672.8 (s), 510.1 (s) cm^−1^. LC-MS (ESI) 283.0 (M + H). HR-MS (ESI) calculated for C_16_H_12_N_2_OCl 283.0683, found 283.0645 (Δ = 2.5 ppm). Melting range: 152.6–155.0 °C.

*(E)-1-((3-Methylphenyl)diazenyl)naphthalene-2-ol* (**29**) [83,86]: Assessed loading 18.1 mmol, 87%; Isolated 4.47 g, 82%. ^1^H-NMR (400 MHz, DMSO-*d*_6_) δ/ppm: 15.83 (s, 1H), 8.55 (d, *J* = 7.8 Hz, 1H), 7.95 (d, *J* = 9.4 Hz, 1H), 7.79 (dd, *J* = 1.3, 7.8 Hz, 1H), 7.69 (s, 1H), 7.60-7.66 (m, 2H), 7.40–7.50 (m, 2H), 7.20 (d, *J* = 7.8 Hz, 1H), 6.93 (d, *J* = 9.4 Hz, 1H), 2.42 (s, 3H). ^13^C-NMR (101 MHz, DMSO-*d*_6_) δ/ppm: 169.2 (C), 145.5 (C), 140.3 (CH), 139.9 (C), 133.2 (C), 130.1 (CH), 129.6 (C), 129.5 (CH), 129.4 (CH), 129.3 (CH), 128.3 (C), 126.2 (CH), 124.4 (CH), 121.8 (CH), 119.6 (CH), 116.8 (CH), 21.5 (CH_3_). IR (neat) ν 3030 (w), 2920 (w), 1616 (m), 1505 (s), 1447 (s), 1271 (m), 1236 (s), 1209 (s), 1126 (s), 986 (m), 864 (s), 835 (s), 780 (s), 748 (s), 682 (s), 513 (s) cm^−1^. LC-MS (ESI) 263.1 (M + H). HR-MS (ESI) calculated for C_17_H_15_N_2_O 263.1184, found 263.1183 (Δ = 0.4 ppm). Melting range: 138.1–139.0 °C.

*(E)-1-((2-Methyl-5-nitrophenyl)diazenyl)naphthalene-2-ol* (**30**) [87]: Assessed loading 5.9 mmol, 67%; Isolated 1.98 g, 60%. ^1^H-NMR (400 MHz, CDCl_3_) δ/ppm: 8.76 (1H, d, *J* = 2.4 Hz), 8.50 (1H, d, *J* = 8.1 Hz), 7.95 (1H, dd, *J* = 8.2, 2.4 Hz), 7.69 (1H, d, *J* = 9.5 Hz), 7.59 (1H, d, *J* = 8.0 Hz), 7.54 (1H, d, *J* = 7.8 Hz), 7.44 (1H, t, *J* = 7.2 Hz), 7.38 (1H, d, *J* = 8.0 Hz), 6.75 (1H, d, *J* = 9.5 Hz), 2.56 (3H, s). ^13^C-NMR (101 MHz, CDCl_3_) δ/ppm: 176.5 (C), 147.8 (C), 142.8 (C), 142.3 (CH), 134.1 (C), 133.0 (C), 131.7 (CH+C), 129.7 (CH), 128.9 (CH), 128.3 (C), 126.9 (CH), 125.4 (CH), 122.2 (CH), 120.0 (CH), 110.2 (CH), 17.8 (CH_3_). IR (neat) ν 1611.2 (m), 1523.2 (s), 1498.9 (s), 1448.4 (m), 1342.9 (s), 1274.3 (m), 1194.9 (s), 1154.7 (m), 1135.0 (m), 841.2 (s), 795.6 (s), 759.7 (s), 736.5 (s), 507.0 (m) cm^−1^. LC-MS (ESI) 375.0 (M + H). HR-MS (ESI) calculated for C_17_H_14_N_3_O_3_ 308.1035, found 308.1041 (Δ = −1.3 ppm). Melting range: 208.3–210.6 °C.

*(E)-1-((2-Nitrophenyl)diazenyl)naphthalene-2-ol* (**31**) [88]: Assessed loading 5.8 mmol, 82%; Isolated 1.60 g, 77%. ^1^H-NMR (400 MHz, CDCl_3_) δ/ppm: 8.42 (1H, dd, *J* = 8.6, 1.3 Hz), 8.38 (1H, d, *J* = 8.0 Hz), 8.29 (1H, dd, *J* = 8.5, 1.5 Hz), 7.73 (1H, dddd, *J* = 8.6, 7.2, 1.5, 0.7 Hz), 7.63 (1H, d, *J* = 9.7 Hz), 7.52 (1H, ddd, *J* = 8.2, 7.1, 1.5 Hz), 7.48 (1H, dd, *J* = 7.7, 1.5 Hz), 7.41 (1H, ddd, *J* = 7.7, 7.1, 1.2 Hz,), 7.23 (1H, ddd, *J* = 8.5, 7.1, 1.3 Hz), 6.68 (1H, d, *J* = 9.7 Hz). ^13^C-NMR (101 MHz, CDCl_3_) δ/ppm: 181.2 (C), 143.4 (CH), 139.3 (C), 135.6 (CH), 133.2 (C), 133.0 (C), 129.6 (CH), 129.2 (CH), 129.0 (C), 127.9 (CH), 127.2 (CH), 126.1 (CH), 123.6 (CH), 123.0 (CH), 117.8 (CH), 100.0 (C). IR (neat) ν 1737.1 (w), 1603.7 (m), 1570.2 (m), 1476.9 (s), 1448.5 (m), 1187.1 (m), 1130.1 (m), 843.5 (m), 738.7 (s), 505.8 (s) cm^−1^. LC-MS (ESI) 294.1 (M + H). HR-MS (ESI) calculated for C_16_H_12_N_3_O_3_ 294.0879, found 294.0883 (Δ = 1.4 ppm). Melting range: 207.0–209.3 °C.

*(E)-1-((4-Nitrophenyl)diazenyl)naphthalene-2-ol* (**32**) [89]: Assessed loading 5.1 mmol, 78%; Isolated 1.30 g, 68%. ^1^H-NMR (400 MHz, CDCl_3_) δ/ppm: δ 16.12 (1H, s), 8.40 (1H, d, *J* = 8.1 Hz), 8.31 (2H, d, *J* = 9.1 Hz), 7.69 (1H, d, *J* = 9.6 Hz), 7.68 (2H, d, *J* = 9.4 Hz,), 7.59–7.50 (2H, m), 7.46–7.39 (1H, m), 6.69 (1H, d, *J* = 9.7 Hz). ^13^C-NMR (101 MHz, CDCl_3_) δ/ppm: 180.3 (C), 147.9 (C), 144.5 (C), 143.5 (CH), 133.0 (C), 132.0 (C), 129.8 (CH), 129.2 (CH), 128.6 (C), 127.6 (CH), 126.4 (CH), 125.7 (2CH), 122.5 (CH), 116.6 (2CH). IR (neat) ν 1739.7 (w), 1591.7 (m), 1498.7 (s), 1329.9 (s), 1226.2 (m), 1202.4 (s), 1153.6 (m), 1106.2 (s), 859.6 (m), 835.4 (s), 747.7 (s), 488.9 (s) cm^−1^. LC-MS (ESI) 294.1 (M + H). HR-MS (ESI) calculated for C_16_H_12_N_3_O_3_ 294.0879, found 294.0881 (Δ = 0.7 ppm). Melting range: 250.3–253.1 °C.

*(E)-1-((4-Bromophenyl)diazenyl)naphthalene-2-ol* (**33**) [90,91,92]: Assessed loading 11.3 mmol, 90%; Isolated 3.52 g, 86%. ^1^H-NMR (400 MHz, CDCl_3_) δ/ppm: 16.06 (1H, s), 8.56–8.50 (1H, d, *J* = 8.4 Hz), 7.73 (1H, d, *J* = 9.5 Hz), 7.60–7.68 (1H, m,), 7.60 (4H, m), 7.56 (1H, ddd, *J* = 8.3, 7.3, 1.5 Hz), 7.44–7.37 (1H, m), 6.86 (1H, d, *J* = 9.4 Hz,). ^13^C-NMR (101 MHz, CDCl_3_) δ/ppm: 171.4 (C), 144.1 (C), 140.3 (CH), 133.4 (C), 132.7 (2CH), 130.3 (C), 129.0 (CH), 128.7 (CH), 128.2 (C), 126.0 (CH), 124.5 (CH), 121.8 (CH), 120.8 (C), 120.0 (2CH). IR (neat) ν 1737.5 (w), 1617.3 (w), 1476.9 (m), 1252.3 (m), 1209.2 (m), 1071.8 (m), 1004.8 (m), 817.7 (s), 748.8 (s), 494.9 (s) cm^−1^. LC-MS (ESI) 327.0 (M + H). HR-MS (ESI) calculated for C_16_H_12_N_2_OBr 327.0133, found 327.0126 (Δ = −2.1 ppm). Melting range: 157.8–160.3 °C.

*(E)-1-(Phenyldiazenyl)naphthalene-2-ol* (**34**) [93,94]: Assessed loading 16.7 mmol, 90%; Isolated 3.86 g, 83%. ^1^H-NMR (400 MHz, DMSO-*d*_6_) δ/ppm: 15.76 (s, 1H), 8.52 (d, *J* = 8.2 Hz, 1H), 7.93 (d, *J* = 9.4 Hz, 1H), 7.84 (d, *J* = 8.2 Hz, 2H), 7.76 (d, *J* = 8.2 Hz, 1H), 7.60 (ddd, *J* = 1.4, 7.0, 8.4 Hz, 1H), 7.54 (t, *J* = 8.2 Hz, 2H), 7.44 (ddd, *J* = 1.0, 1.2, 7.1 Hz, 1H), 7.38 (t, *J* = 8.2 Hz, 1H), 6.90 (d, *J* = 9.4 Hz, 1H). ^13^C-NMR (101 MHz, DMSO-*d*_6_) δ/ppm: 169.3 (C), 145.5 (C), 140.4 (CH), 133.2 (C), 130.2 (2CH), 129.6 (C), 129.5 (CH), 129.3 (CH), 128.5 (CH), 128.3 (C), 126.3 (CH), 124.3 (CH), 121.7 (CH), 119.4 (2CH). IR (neat) ν 3035 (w), 1616 (m), 1496 (s), 1447 (s), 1387 (m), 1254 (s), 1205 (s), 1135 (s), 984 (s), 841 (s), 749 (s), 682 (s), 493 (s) cm^−1^. LC-MS (ESI) 249.2 (M + H). HR-MS (ESI) calculated for C_16_H_13_N_2_O 249.1028, found 249.1032 (Δ = 1.6 ppm). Melting range: 127.9–130.2 °C.

*(E)-1-((4-Chlorophenyl)diazenyl)naphthalene-2-ol* (**35**): Assessed loading 12.2 mmol, 94%; Isolated 3.11 g, 85%. ^1^H-NMR (400 MHz, CDCl_3_) δ/ppm 16.04 (1H, s), 8.54 (1H, dd, *J* = 8.2, 1.2 Hz), 7.73 (1H, d, *J* = 9.4 Hz), 7.67 (2H, d, *J* = 8.8 Hz), 7.61 (1H, d, *J* = 8.0 Hz), 7.56 (1H, ddd, *J* = 8.4, 7.1, 1.3 Hz), 7.47–7.36 (3H, m), 6.88 (1H, d, *J* = 9.4 Hz). ^13^C-NMR (101 MHz, CDCl_3_) δ/ppm: 170.7 (C), 143.8 (C), 140.1 (CH), 133.4 (C), 133.0 (C), 130.2 (C), 129.7 (2CH), 128.9 (CH), 128.7 (CH), 128.2 (C), 125.9 (CH), 124.3 (CH), 121.7 (CH), 119.9 (2CH). IR (neat) ν 1617.3 (w), 1483.9 (m), 1253.5 (m), 1210.7 (m), 1089.8 (m), 820.3 (s), 748.7 (s), 497.1 (s) cm^−1^. LC-MS (ESI) 283.0 (M + H). HR-MS (ESI) calculated for C_16_H_12_N_2_OCl 283.0638, found 283.0644 (Δ = 2.1 ppm). Melting range: 153.2–156.0 °C.

*(E)-1-(Benzo[d][1,3]dioxol-5-yldiazenyl)naphthalene-2-ol* (**36**): Assessed loading 20.2 mmol, 83%; Isolated 5.40 g, 76%. ^1^H-NMR (400 MHz, CDCl_3_) δ/ppm: 15.50 (1H, s), 8.70 (1H, d, *J* = 8.4 Hz), 7.77 (1H, dt, *J* = 9.1, 0.6 Hz), 7.70 (1H, d, *J* = 8.0 Hz,), 7.58 (1H, ddd, *J* = 8.4, 7.0, 1.3 Hz), 7.51 (1H, d, *J* = 2.0 Hz), 7.41 (1H, ddd, *J* = 8.1, 7.0, 1.3 Hz), 7.32 (1H, dd, *J* = 8.2, 2.0 Hz), 7.06 (1H, d, *J* = 9.2 Hz), 6.92 (1H, d, *J* = 8.2 Hz), 6.07 (2H, s). ^13^C-NMR (101 MHz, CDCl_3_) δ/ppm: 160.4 (C), 149.2 (C), 149.0 (C), 143.8 (C), 136.7 (CH), 133.2 (C), 129.5 (C), 128.3 (CH), 128.21 (C), 128.2 (CH), 124.9 (CH), 121.9 (CH), 121.6 (CH), 118.7 (CH), 108.4 (CH), 102.0 (CH_2_), 98.6 (CH). IR (neat) ν 2899.5 (w), 1621.2 (m), 1499.6 (m), 1476.8 (s), 1457.3 (s), 1036.9 (s), 820.6 (m), 750.0 (m) cm^−1^. LC-MS (ESI) 293.0 (M + H). HR-MS (ESI) calculated for C_17_H_13_N_2_O_3_ 293.0926, found 293.0934 (Δ = 2.7 ppm). Melting range: 143.7–146.2 °C.

## 4. Conclusions

We have evaluated a series of diazonium forming reactions performed using flow chemistry to aid in the production of these important and synthetically versatile salts. The use of solid supported acids in flow was shown as a viable approach should small quantities of isolated material be required (~50 mg −1 g). Importantly the electronics of the parent aniline played an important role in the efficiency of the methodology and indicated its preferential use for more electron rich aniline starting materials. For the generation of larger quantities of material expedience intrinsically directs the synthetic methodology to more solution phase and continuous production operation. In such cases the use of aqueous conditions may be hindered by solubility limitations of both the starting anilines and resultant diazonium intermediates, a general working range of 0.3 + 0.26 M was determined for flow. Beneficially flow proved advantageous for exerting control over mixing and to easily regulate temperature to inhibit deleterious side reactions such as phenol formation, a temperature operational working window was defined. Alternatively organic nitrite donors were shown be useful in organic solvents to easily prepare diazonium intermediates. Of note no additional acid activator was required for these transformations although trimethyl silyl halides could be added to produce in situ nitrosyl halides as that can be used as diazonium forming species.

In general a wide range of preparation conditions and the use of several diazotising agents have been successfully demonstrated in flow. We believe the production of these valuable diazonium intermediates as a continuous stream which as shown can be intercepted to create additional derivatives adds significant value. The elimination of intermediate isolation and handling of diazonium species improves both the overall safety of the process and reduces potential risks due to health hazards associated with such compounds. In addition, once optimised scale up or repeated access to quantities of these species can be automated saving valuable synthesis time which can be employed on more challenging endeavours.

## Figures and Tables

**Table 1:** Flow in situ formation of hydrazine derivatives from diazonium salts.

Product ^A^	Aniline Substrate	Yield (%)
**1**	2-Br	79
**2**	3-Br	80
**3**	4-Br	94
**4**	2-Cl	68
**5**	3-Cl	83
**6**	4-Cl	90
**7**	2-NO_2_	89
**8**	3-NO_2_	92
**9**	4-NO_2_	90
**10**	2-OMe	67
**11**	3-Me	72
**12**	4-Me	77

^A^: The products were isolated by basification of the reaction mixture pH ~ 8 followed by extraction with EtOAc. The aqueous solution was then acidified to pH ~ 4 and extracted with EtOAc, the organic phase was dried over MgSO_4_, the solvent evaporated and the isolated compound characterized.

**Table 2:** Loading results for the supported acids using PhNH_2_ in MeCN.

Supported Acid	Theoretical Loading (mmol/g)	Measured Loading (mmol/g)
MP-TOSH	4.45	3.55 ^A^/3.84 ^B^
Si-SA (SCX)	0.63	0.61/0.62 ^C^/0.60 ^D^
Si-SA (SCX2)	0.54	0.53/0.53 ^C^/0.53 ^D^
Nafion NR50	0.80	0.37 ^A^/0.54 ^B^/0.77 ^E^
Nafion NR50^F^	0.80	0.66

A standard 6 g of supported acid was used in a glass column (10 cm length, 6.6 mm i.d. with adjustable length end pieces), 1 equivalent of aniline was passed through the resin followed by washing by 5 column volumes of pure solvent, results given for MeCN. ^A^: 0.25 mL/min; ^B^: 0.10 mL/min; ^C^: 0.5 mL/min; ^D^: 1 mL/min; ^E^: 3 h in batch; ^F^: The material was crushed into a powder and mixed with 20% *w*/*w* MgSO_4_.

**Table 3:** Loading results for 6 g of MP-TsOH using different aniline starting materials dissolved in EtOH @ 0.50 mL/min.

Product	Aniline Substrate	Loading Time (min)	Processed Aniline (mmol) ^A^	Loading Efficiency (%) ^B^	Diazo Dye Formation mmol, (%) ^C^
**20**	3-F	32.0	16.0	60	14.2, 89
**21**	3-CF_3_	29.6	14.8	55	12.7, 86
**22**	4-CN	20.0	10.0	37	9.3, 93
**23**	3-OMe	47.0	23.5	87	21.3, 91
**24**	4-OMe	51.4	25.7	95	22.9, 89
**25**	2-F,4-CN	26.8	13.4	50	11.6, 87
**26**	2,4-F	24.4	12.2	46	11.0, 90
**27**	2-Cl,5-OPh	33.8	16.9	63	nd ^D^
**28**	3-Cl	33.2	16.6	62	14.3, 86
**29**	3-Me	41.8	20.9	77	18.1, 87
**30**	2-Me,5-NO_2_	18.6	8.8	33	5.9, 67
**31**	2-NO_2_	14.2	7.1	26	5.8, 82
**32**	4-NO_2_	12.8	6.4	24	4.4, 69
**32**	4-NO_2_	13.0	6.5	25	5.1, 78 ^E^
**33**	4-Br	25.2	12.6	47	11.3, 90
**34**	H	37.2	18.6	89	16.7, 90
**35**	4-Cl	26.0	13.0	58	12.2, 94
**36**	Dioxol-5yl	48.4	24.2	81	20.2, 83

^A^: Amount of aniline processed before breakthrough was detected by in-line MS analysis; ^B^: Calculated as theoretical loading divided by actual loading multiplied by 100; ^C^: Percentage conversion based on aniline loading; ^D^: compound crystalized in the column; ^E^: The column was washed for a further 20 min with EtOH to elute more of the product from the column.

## References

[1] Yoshida J., Takahashi Y., Nagaki A. (2013). Flash chemistry: Flow chemistry that cannot be done in batch. Chem. Commun..

[2] Gutmann B., Kappe C.O. (2015). Forbidden Chemistries go flow in API. Chim. Oggi Chem. Today.

[3] Baxendale I.R. (2013). The integration of flow reactors into synthetic organic chemistry. J. Chem. Technol. Biotechnol..

[4] Shang M., Noël T., Wang Q., Su Y., Miyabayashi K., Hessel V., Hasebe S. (2015). 2- and 3-Stage temperature ramping for the direct synthesis of adipic acid in micro-flow packed-bed reactors. Chem. Eng. J..

[5] Snead D.R., Jamison T.F. (2015). A Three-Minute Synthesis and Purification of Ibuprofen: Pushing the Limits of Continuous-Flow Processing. Angew. Chem. Int. Ed..

[6] van den Broek S.A.M.W., Leliveld J.R., Becker R., Delville M.M.E., Nieuwland P.J., Koch K., Rutjes F.P.J.T. (2012). Continuous Flow Production of Thermally Unstable Intermediates in a Microreactor with Inline IR-Analysis: Controlled Vilsmeier–Haack Formylation of Electron-Rich Arenes. Org. Process Res. Dev..

[7] Hafner A., Ley S.V. (2015). Generation of Reactive Ketenes under Flow Conditions through Zinc-Mediated Dehalogenation. Synlett.

[8] Porta R., Benaglia M., Puglisi A. (2016). Flow Chemistry: Recent Developments in the Synthesis of Pharmaceutical Products. Org. Process Res. Dev..

[9] Baumann M., Baxendale I.R. (2015). The synthesis of active pharmaceutical ingredients (APIs) using continuous flow chemistry. Beilstein J. Org. Chem..

[10] Gutmann B., Cantillo D., Kappe C.O. (2015). Continuous-flow technology—A tool for the safe manufacturing of active pharmaceutical ingredients. Angew. Chem. Int. Ed..

[11] Denčić I., Ott D., Kralisch D., Noël T., Meuldijk J., de Croon M., Hessel V., Laribi Y., Perrichon P. (2014). Continuous Processing in the Manufacture of Active Pharmaceutical Ingredients and Finished Dosage Forms: An Industry Perspective. Org. Process Res. Dev..

[12] Newman S.G., Jensen K.F. (2013). The role of flow in green chemistry and engineering. Green Chem..

[13] Malet-Sanz L., Susanne F. (2012). Continuous Flow Synthesis. A Pharma Perspective. J. Med. Chem..

[14] Anderson N.G. (2001). Practical Use of Continuous Processing in Developing and Scaling Up Laboratory Processes. Org. Process Res. Dev..

[15] Oger N., Le Grognec E., Felpin F.-X. (2015). Handling diazonium salts in flow for organic and material chemistry. Org. Chem. Front..

[16] Oger N., d’Halluin M., Le Grognec E., Felpin F.-X. (2014). Aryldiazonium Tetrafluoroborate Salts as Green and Efficient Coupling Partners for the Suzuki–Miyaura Reaction: From Optimisation to Mole Scale. Org. Process Res. Dev..

[17] Smith C.J., Nikbin N., Ley S.V., Lange H., Baxendale I.R. (2011). A fully automated, multistep flow synthesis of 5-amino-4-cyano-1,2,3-triazoles. Org. Biomol. Chem..

[18] Browne D.L., Baxendale I.R., Ley S.V. (2011). Piecing together the puzzle: understanding a mild, metal free reduction method for the large scale synthesis of hydrazines. Tetrahedron.

[19] Smith C.J., Nikbin N., Smith C.D., Ley S.V., Baxendale I.R. (2011). Flow synthesis of organic azides and the multistep synthesis of imines and amines using a new monolithic triphenylphosphine reagent. Org. Biomol. Chem..

[20] Malet-Sanz L., Madrzak J., Ley S.V., Baxendale I.R. (2010). Preparation of arylsulfonyl chlorides by chlorosulfonylation of in situ generated diazonium salts using a continuous flow reactor. Org. Biomol. Chem..

[21] Yu Z., Xie X., Dong H., Liu J., Su W. (2016). Continuous-Flow Process for the Synthesis of m-Nitrothioanisole. Org. Process Res. Dev..

[22] Deadman B.J., Collins S.G., Maguire A.R. (2015). Taming Hazardous Chemistry in Flow: The Continuous Processing of Diazo and Diazonium Compounds. Chem. Eur. J..

[23] Tran D.N., Battilocchio C., Lou S.-B., Hawkins J.M., Ley S.V. (2015). Flow chemistry as a discovery tool to access sp^2^–sp^3^ cross-coupling reactions via diazo compounds. Chem. Sci..

[24] Yu Z., Tong G., Xie X., Zhou P., Lv Y., Su W. (2015). Continuous-Flow Process for the Synthesis of 2-Ethylphenylhydrazine Hydrochloride. Org. Process Res. Dev..

[25] Nalivela K.S., Tilley M., McGuire M.A., Organ M.G. (2014). Multicomponent, Flow Diazotization/Mizoroki–Heck Coupling Protocol: Dispelling Myths about Working with Diazonium Salts. Chem. Eur. J..

[26] Oger N., Le Grognec E., Felpin F.-X. (2014). Continuous-Flow Heck–Matsuda Reaction: Homogeneous versus Heterogeneous Palladium Catalysts. J. Org. Chem..

[27] Yu Z., Lv Y., Yu C., Su W. (2013). Continuous flow reactor for Balz–Schiemann reaction: A new procedure for the preparation of aromatic fluorides. Tetrahedron Lett..

[28] Chen M., Buchwald S.L. (2013). Continuous-Flow Synthesis of 1-Substituted Benzotriazoles from Chloronitrobenzenes and Amines in a C-N Bond Formation/Hydrogenation/Diazotization/Cyclization Sequence. Angew. Chem. Int. Ed..

[29] Delville M.M.E., van Hest J.C.M., Rutjes F.P.J.T. (2013). Ethyl diazoacetate synthesis in flow. Beilstein J. Org. Chem..

[30] Li B., Widlicka D., Boucher S., Hayward C., Lucas J., Murray J.C., O’Neil B.T., Pfisterer D., Samp L., van Alsten J. (2012). Telescoped Flow Process for the Syntheses of *N*-Aryl Pyrazoles. Org. Process Res. Dev..

[31] Yu Z., Lv Y., Yu C. (2012). A Continuous Kilogram-Scale Process for the Manufacture of o-Difluorobenzene. Org. Process Res. Dev..

[32] Hu D.X., O’Brien M., Ley S.V. (2012). Continuous Multiple Liquid–Liquid Separation: Diazotization of Amino Acids in Flow. Org. Lett..

[33] Kuznetsov V.V., Zemyatova S.V. (2007). Flow-injection spectrophotometry of nitrites based on the diazotization reactions of azine dyes. J. Anal. Chem..

[34] Behringer H., Karrenbauer K. (1981). Continuous Diazotization of Amines. U.S. Patent.

[35] Wootton R.C.R., Fortt R., de Mello A.J. (2002). On-chip generation and reaction of unstable intermediates—Monolithic nanoreactors for diazonium chemistry: Azo dyes. Lab. Chip.

[36] Guenther P.M., Moeller F., Henkel T., Koehler J.M., Groβ G.A. (2005). Formation of Monomeric and Novolak Azo Dyes in Nanofluid Segments by Use of a Double Injector Chip Reactor. Chem. Eng. Technol..

[37] Fortt R., Wootton R.C.R., de Mello A.J. (2003). Continuous-Flow Generation of Anhydrous Diazonium Species: Monolithic Microfluidic Reactors for the Chemistry of Unstable Intermediates. Org. Process Res. Dev..

[38] Carter C.C., Lange H., Ley S.V., Baxendale I.R., Wittkamp B., Goode J.G., Gaunt N.L. (2010). ReactIR Flow Cell: A New Analytical Tool for Continuous Flow Chemical Processing. Org. Process Res. Dev..

[39] Carter C.C., Lange H., Ley S.V., Baxendale I.R. (2010). The Continuous Flow Synthesis of Butane-2,3-Diacetal Protected Building Blocks Using Microreactors. Org. Biomol. Chem..

[B40-molecules-21-00918] 40.Either the commercial hydrochloride salt was bought or the salt was prepared by the addition of one equivalent of concentrated hydrochloric acid (37%) to the aqueous solution and the mixture sonicated until the aniline fully dissolved.

[B41-molecules-21-00918] 41.Characterized by cleaner visual reaction streams comprising of non-brown homogenous mixtures, the elimination of solution cloudiness and significant ppt or oil formation at higher concentrations. By analysis less by-product formation {diazo dye formation <2% N=N signal at ~2250}.

[42] Westcott B., Enemark J., Soloman E., Lever A. (1999). Inorganic Electronic Structure and Spectroscopy.

[43] Wiseman F.L. (2005). Monitoring the Rate of Solvolytic Decomposition of Benzenediazonium Tetrafluoroborate in Aqueous Media Using a pH Electrode. J. Chem. Educ..

[44] Taylor J.E., Feltis T.J. (1952). The Hydrolytic Decomposition of Diazonium Salts. I. The Determination of Very Precise Rates in Very Dilute Solutions. J. Am. Chem. Soc..

[45] Canning P.S.J., McCrudden K., Masnill H., Sexton B. (1999). Rates and mechanisms of the thermal solvolytic decomposition of arenediazonium ions. J. Chem. Soc. Perkin Trans..

[46] Smith B.D., Cox J.R. (1966). The Investigation of the Decomposition of Diazonium Salts in Aqueous Solution.

[47] Broxton T.J., McLeish M.J. (1982). Reactions of aryl diazonium salts and arylazo alkyl ethers. VI. A comparison of the available methods for the measurement of the rate of ionization of (*Z*)-arylazo alkyl ethers in alcoholic solvents. Aust. J. Chem..

[48] Moelwyn-Hugh E.A., Johnson P. (1940). The kinetics of the decomposition of benzene diazonium chloride in water. Trans. Faraday Soc..

[49] Hegarty A.F., Patai S. (1978). Kinetics and mechanisms of reactions involving diazonium and diazo groups. Diazonium and Diazo Groups.

[50] Polar Bear Plus Reactor Available from Cambridge Reactor Design. http://www.cambridgereactordesign.com/polarbearplus/index.html.

[51] Uniqsis Glass Static Mixer-Reactor Chips. http://www.uniqsis.com/.

[52] Poh J.-S., Browne D.L., Ley S.V. (2016). A multistep continuous flow synthesis machine for the preparation of pyrazoles via a metal-free amine-redox process. React. Chem. Eng..

[53] Ashcroft C.P., Hellier P., Pettman A., Watkinson S. (2011). Second-Generation Process Research towards Eletriptan: A Fischer Indole Approach. Org. Process Res. Dev..

[54] Norris T., Bezze C., Franz S.Z., Stivanello M. (2009). Heavy-Metal-Free Reduction Methodology for Large-Scale Synthesis Applicable to Heterocyclic and Arylhydrazines. Org. Process Res. Dev..

[55] Doyle M.P., Nesloney C.L., Shanklin M.S., Marsh C.A., Brown K.C. (1989). Formation and characterization of 3-O-arenediazoascorbic acids. New stable diazo ethers. J. Org. Chem..

[56] Weiss R., Wagner K.-G. (1984). Notizen. Die Erzeugung von Nitrosylsalzen in wasserfreien organischen Medien. Chem. Ber..

[57] Colas C., Goeldner M. (1999). An Efficient Procedure for the Synthesis of Crystalline Aryldiazonium Trifluoroacetates—Synthetic Applications. Eur. J. Org. Chem..

[58] Mallia C.J., Baxendale I.R. (2016). The Use of Gases in Flow Synthesis. Org. Process Res. Dev..

[59] Koos P., Gross U., Polyzos A., O’Brien M., Baxendale I.R., Ley S.V. (2011). Teflon AF-2400 mediated gas–liquid contact in continuous flow methoxycarbonylations and in-line FTIR measurement of CO concentration. Org. Biomol. Chem..

[60] O’Brien M., Taylor N., Polyzos A., Baxendale I.R., Ley S.V. (2011). Hydrogenation in flow: Homogeneous and heterogeneous catalysis using Teflon AF-2400 to effect gas–liquid contact at elevated pressure. Chem. Sci..

[61] O’Brien M., Baxendale I.R., Ley S.V. (2010). Flow Ozonolysis Using a Semipermeable Teflon AF-2400 Membrane To Effect Gas−Liquid Contact. Org. Lett..

[62] Baxendale I.R., Mallia C.J., Brocken L. (2013). Flow chemistry approaches directed at improving chemical synthesis. Green Process Synth..

[63] Baumann M., Baxendale I.R., Ley S.V. (2011). The flow synthesis of heterocycles for natural product and medicinal chemistry applications. Mol. Divers..

[64] Chaturbhuj G.U., Akamanchi K.G. (2011). Copper catalyzed Gomberg–Buchmann–Hey reaction using aryldiazonium tosylate. Tetrahedron Lett..

[65] Krasnokutskaya E.A., Semenischeva N.I., Filimonov V.D., Knochel P. (2007). A New, One-Step, Effective Protocol for the Iodination of Aromatic and Heterocyclic Compounds via Aprotic Diazotization of Amines. Synthesis.

[66] Lu Y.-T., Arai C., Ge J.-F., Ren W.-S., Kaiser M., Wittlin S., Brun R., Lu J.-M., Ihara M. (2011). Synthesis and in vitro antiprotozoal activities of water-soluble, inexpensive phenothiazinium chlorides. Dyes Pigment..

[67] Zarei A., Khazdooz L., Pirisedigh A., Hajipour A.R., Seyedjamali H., Aghaei H. (2011). Aryldiazonium silica sulfates as efficient reagents for Heck-type arylation reactions under mild conditions. Tetrahedron Lett..

[68] Filimonov V.D., Trusova M., Postnikov P., Krasnokutskaya E.A., Lee Y.M., Hwamg H.Y., Kim H., Chi K.-W. (2008). Unusually Stable, Versatile, and Pure Arenediazonium Tosylates: Their Preparation, Structures, and Synthetic Applicability. Org. Lett..

[69] Caldarelli C., Baxendale I.R., Ley S.V. (2000). Clean and efficient synthesis of azo dyes using polymer-supported reagents. Green Chem..

[70] MP-TsOH Sulfonic Acid Resin Is Available from Biotage AB (Part Number 800463). http://www.biotage.com.

[71] SiliaBond^®^ Propylsulfonic Acid (SCX-2) and SiliaBond^®^ Tosic Acid (SCX). http://www.silicycle.com.

[B72-molecules-21-00918] 72.Nafion^®^ NR50, CAS Number 31175–20–9 (loading 0.8 meq/g) is available from Sigma Aldrich. (accessed on 13 July 2016).

[73] Zak J., Ron D., Riva E., Harding H.P., Cross B.C.S., Baxendale I.R. (2012). Establishing a Flow Process to Coumarin-8-Carbaldehydes as Important Synthetic Scaffolds. Chem. Eur. J..

[74] Browne D.L., Wright S., Deadman B., Dunnage S., Baxendale I.R., Turner R., Ley S.V. (2012). Continuous flow reaction monitoring using an on-line miniature mass spectrometer. Rapid Commun. Mass Spectrom..

[75] Holmes N., Akien G.R., Savage R.J.D., Stanetty C., Baxendale I.R., Blacker A.J., Taylor B.A., Woodward R.L., Meadows R.E., Bourne R.A. (2016). Online quantitative mass spectrometry for the rapid adaptive optimisation of automated flow reactors. React. Chem. Eng..

[76] Guthrie J.P. (1978). Hydrolysis of esters of oxy acids: pKa values for strong acids; Brønsted relationship for attack of water at methyl; free energies of hydrolysis of esters of oxy acids; and a linear relationship between free energy of hydrolysis and pKa holding over a range of 20 pK units. Can. J. Chem..

[77] Eckert F., Leito I., Kaljurand I., Kütt A., Klamt A., Diedenhofen M. (2009). Prediction of acidity in acetonitrile solution with COSMO-RS. J. Comput. Chem..

[78] Kütt A., Ivo Leito I., Kaljurand I., Sooväli L., Vlasov V.M., Yagupolskii L.M., Koppel I.A. (2006). A Comprehensive Self-Consistent Spectrophotometric Acidity Scale of Neutral Brønsted Acids in Acetonitrile. J. Org. Chem..

[79] Lee L., Leroux Y.R., Hapiot P., Downard A.J. (2015). Amine-Terminated Monolayers on Carbon: Preparation, Characterization, and Coupling Reactions. Langmuir.

[80] Cartwright R.A., Tatlow J.C. (1953). The reactions of certain nitrogen-containing compounds derived from benzotrifluoride. J. Chem. Soc..

[81] Chen S., Tsao M.-L. (2013). Genetic Incorporation of a 2-Naphthol Group into Proteins for Site-Specific Azo Coupling. Bioconjugate Chem..

[82] Yamada T., Tanaka N., Morisawa T., Nishikuri M., Kaji A. (1970). Studies of Diazosulfides. I. A Kinetic Study of the Reaction of Diaryldiazosulfides with β-Naphthol in Alkaline Ethanol. Bull. Chem. Soc. Jpn..

[83] Mohamed S.K., Gomaa M.A.-M., Nour El-Din A.M. (1997). Reaction of Triazene 1-Oxides: Novel Synthesis of Solid Arenediazonium Chlorides. J. Chem. Res..

[84] Barbero M., Crisma M., Degani I., Fochi R., Perracino P. (1998). New Dry Arenediazonium Salts, Stabilized to an Exceptionally High Degree by the Anion of o-Benzenedisulfonimide. Synthesis.

[85] Zhang H., Zhou N., Zhu X., Chen X., Zhang Z. (2012). Cyclic Side-Chain Phenylazo Naphthalene Polymers: Enhanced Fluorescence Emission and Surface Relief Grating Formation. Macromol. Rapid Commun..

[86] Rahimizadeh M., Eshghi H., Shiri A., Ghadamyari Z., Matin M.M., Oroojalian F., Pordeli P. (2012). Fe(HSO_4_)_3_ as an Efficient Catalyst for Diazotization and Diazo Coupling Reactions. J. Korean Chem. Soc..

[87] Tedder J.M., Theaker G. (1957). The direct introduction of the diazonium group into aromatic nuclei. Part II. Diazonium salts from aromatic sulphonic acids, carboxylic acids, and nitro-compounds prepared by use of mercuric ions as catalyst. J. Chem. Soc..

[88] Churkina L.N., Belyaev E.Y., Kazak Y.Y. (2001). Synthesis of Dyes from Aromatic C-Nitroso-*N*-hydroxytriazenes. Russ. J. Org. Chem..

[89] Zarchi M.A.K., Karimi M. (2012). Diazotization of anilines and diazo coupling with a coupling component mediated by a polymer-supported sodium nitrite and a polymeric acid. J. Appl. Polym. Sci..

[90] Gorelik M.V., Lomzakova V.I., Khamidova E.A., Shteiman V.Y., Kuznetsova M.G., Andrievsky A.M. (1995). Regioselective Bromination of Anilines in the Presence of Nitrosonium Hydrogensulfate in Concentrated Sulfuric Acid. Mendeleev Comm..

[91] Chin A., Hung M.-H., Stock L.M. (1981). Reactions of benzenediazonium ions with adenine and its derivatives. J. Org. Chem..

[92] Keirstead K.F. (1953). The Reduction of Aromatic Nitro Compounds Magnesium and Methyl alcohol. Can. J. Chem..

[93] Valizadeh H., Amiri M., Hosseinzadeh F. (2012). Nanoparticles of organosilane-based nitrite ionic liquid immobilized on silica for the diazotization of aniline derivatives and subsequent synthesis of azo dyes. Dyes Pigments.

[94] Di Donna L., Maiuolo L., Mazzotti F., de Luca D., Sindona G. (2004). Assay of Sudan I Contamination of Foodstuff by Atmospheric Pressure Chemical Ionization Tandem Mass Spectrometry and Isotope Dilution. Anal. Chem..

